# Supplementary material for: New Polymethoxyflavones from Hottonia palustris Evoke DNA Biosynthesis-Inhibitory Activity in An Oral Squamous Carcinoma (SCC-25) Cell Line
Source: Molecules. 2022 Jul 10;27(14):4415. doi: 10.3390/molecules27144415 (PMC9325269; doi:10.3390/molecules27144415)
Supplement: Supplementary file 1 [file molecules-27-04415-s001.zip › molecules-1784808-supplementary.pdf]

Supplementary Materials

New Polymethoxyflavones from *Hottonia palustris* Evoke DNA Biosynthesis-Inhibitory Activity in An Oral Squamous Carcinoma (SCC-25) Cell Line

Jakub W. Strawa[1], Katarzyna Jakimiuk[1], Łukasz Szoka[2], Krzysztof Brzezinski[3], Paweł Drozdzal[3], Jerzy A. Pałka[2] and Michał Tomczyk[1],*

[1] Department of Pharmacognosy, Faculty of Pharmacy with the Division of Laboratory Medicine, Medical University of Białystok, ul. Mickiewicza 2a, 15-230 Białystok, Poland; jakub.strawa@umb.edu.pl (J.W.S.), katarzyna.jakimiuk@umb.edu.pl (K.J.) michal.tomczyk@umb.edu.pl (M.T.)

[2] Department of Medicinal Chemistry, Faculty of Pharmacy with the Division of Laboratory Medicine, Medical University of Białystok, ul. Mickiewicza 2a, 15-230 Białystok, Poland; lukasz.szoka@umb.edu.pl (L.S.), jerzy.palka@umb.edu.pl (J.P.).

[3] Department of Structural Biology of Prokaryotic Organisms, Institute of Bioorganic Chemistry, Polish Academy of Sciences, ul. Noskowskiego 12/14, 61-074 Poznan, Poland; pdrozdzal@ibch.poznan.pl (P.D.); kbrzezinski@ibch.poznan.pl (K.B.)

* Correspondence: michal.tomczyk@umb.edu.pl; tel.: +48-85-748-56-94

1. Contents

Table S1: Crystal data and structure refinement details for compounds **2** – **7**.

Figure S1: Mass spectrum of compound **4** in negative ion mode (fragmentor = 320 V).

Figure S2: UV spectrum of compound **4**.

Figure S3: ^{1}H NMR spectrum (400 MHz) of compound **4** in $CDCl_3$.

Figure S4: ^{13}C NMR spectrum (100 MHz) of compound **4** in $CDCl_3$.

Figure S5: COSY spectrum of compound **4** in $CDCl_3$.

Figure S6: ROESY spectrum of compound **4** in $CDCl_3$.

Figure S7: HMBC spectrum of compound **4** in $CDCl_3$.

Figure S8: HMQC spectrum of compound **4** in $CDCl_3$.

Figure S9: UV spectrum of compound **5**.

Figure S10: Mass spectrum of compound **5** in negative ion mode (fragmentor = 320 V) .

Figure S11: ^{1}H NMR spectrum (400 MHz) of compound **5** in $CDCl_3$.

Figure S12: ^{13}C NMR spectrum (100 MHz) of compound **5** in $CDCl_3$.

Figure S13: COSY spectrum of compound **5** in $CDCl_3$.

Figure S14: ROESY spectrum of compound **5** in $CDCl_3$.

Figure S15: HSQC spectrum of compound **5** in $CDCl_3$.

Figure S16: HMBC spectrum of compound **5** in $CDCl_3$.

Figure S17: Mass spectrum of compound **6** in negative ion mode (fragmentor = 180 V).

Figure S18: UV spectrum of compound **6**.

Figure S19: ^{1}H NMR spectrum (400 MHz) of compound **6** in DMSO-*d6*.

Figure S20: ^{13}C NMR spectrum (100 MHz) of compound **6** in DMSO-*d6*.

Figure S21: COSY spectrum of compound **6** in DMSO-*d6*.

Figure S22: HMQC spectrum of compound **6** in DMSO-*d6*.

Figure S23: HMBC spectrum of compound **6** in DMSO-*d6*.

Figure S24: ROESY spectrum of compound **6** in DMSO-*d6*.
Figure S25: Mass spectrum of compound **7** in negative ion mode (fragmentor = 180 V).
Figure S26: UV spectrum of compound **7**.
Figure S27: ^{1}H NMR spectrum (400 MHz) of compound **7** in DMSO-*d6*.
Figure S28: ^{13}C NMR spectrum (100 MHz) of compound **7** in DMSO-*d6*.
Figure S29: COSY spectrum of compound **7** in DMSO-*d6*.
Figure S30: ROESY spectrum of compound **7** in DMSO-*d6*.
Figure 31S: HMBC spectrum of compound **7** in DMSO-*d6*.
Figure 32S: HSQC spectrum of compound **7** in DMSO-*d6*.
Table S2: Main HPLC optimization method and validation parameters.
Figure S33: UV–VIS chromatogram of separated compounds **1**-**7** and **ZAP** obtained by HPLC-PDA.
Figure S34: UV–VIS chromatogram of extracts **HP1** and **HP6**-**8** with well-separated compounds **1**-**7** and zapotin (**ZAP**) obtained by HPLC-PDA (345 nm).

Table S1. Crystal data and structure refinement details for compounds **2** – **7**.

Dataset	Compounds					
	**2**	**3**	**4**	**5**	**6**	**7**
Unit cell content	$C_{15}H_{10}O_3$	$C_{16}H_{12}O_4$	$C_{17}H_{14}O_5$	$C_{18}H_{16}O_6$	$C_{16}H_{12}O_5$	$C_{17}H_{14}O_6$
Molecular weight	238.23	268.26	298.28	328.31	284.26	314.28
Temperature [K]	100(2)	100(2)	100(2)	100(2)	100(2)	100(2)
Wavelength [Å]	1.54184	1.54184	1.54184	1.54184	1.54184	1.54184
Crystal system	monoclinic	monoclinic	monoclinic	triclinic	orthorhombic	triclinic
Space group	$P2_1/c$	$P2_1/n$	$P2_1/n$	$P\bar{1}$	$P2_12_12_1$	$P\bar{1}$
Unit cell parameters [Å, °]						
a	4.6496 (1)	7.2599 (1)	10.6669 (1)	7.7919 (2)	11.752 (2)	7.7208 (1)
b	18.7929 (2)	11.4657 (2)	3.9718 (1)	8.9110 (2)	15.486 (3)	15.5433 (1)
c	12.6394 (2)	14.6121 (3)	31.7324 (4)	12.3958 (3)	20.670 (4)	23.6100 (2)
α	90	90	90	101.460 (2)	90	80.347 (1)
β	94.055 (1)	92.207 (2)	93.103 (1)	90.665 (2)	90	85.313 (1)
γ	90	90	90	115.709 (2)	90	82.853 (1)
Volume [Å^3]	1101.67 (2)	1215.41 (4)	1342.42 (3)	755.35 (3)	3761.8 (13)	2766.17 (5)
Z	4	4	4	2	12	8
Absorption coefficient [mm^{-1}]	0.82	0.88	0.91	0.91	0.95	0.97
F(000)	496	560	624	344	1776	1312
Crystal size [mm^3]	0.22×0.20×0.03	0.11×0.03×0.02	0.09×0.02×0.02	0.35×0.03×0.02	0.30×0.04×0.02	0.33×0.02×0.01
θ range for data collection [°]	4.2 to 76.8	4.9 to 76.9	2.8 to 76.9	3.7 to 75.7	3.6 to 72.1	2.9 to 78.8
Index ranges	−5←h←5<br>−23←k←23<br>−15←l←15	−7←h←9<br>−14←k←14<br>−18←l←18	−13←h←13<br>−5←k←4<br>−40←l←39	−8←h←9<br>−10←k←10<br>−15←l←15	−13←h←14<br>−19←k←19<br>−25←l←25	−8←h←9<br>−19←k←19<br>−29←l←29
Reflections collected	18154	12966	26359	7387	73890	112787
Independent reflections, R_{int}	2316, 0.032	2546, 0.030	2814, 0.032	2812, 0.013	7375, 0.074	11577, 0.047
Data / restraints / parameters	2316 / 0 / 167	2546 / 0 / 186	2814 / 0 / 205	2812 / 0 / 224	7375 / 0/ 588	11577 / 0 /862

Goodness-of-fit on F^2	1.07	1.17	1.04	1.08	0.98	1.12
Final R indices (all data)	R1 = 0.035<br>wR2 = 0.098	R1 = 0.052<br>wR2 = 0.148	R1 = 0.034<br>wR2 = 0.094	R1 = 0.038<br>wR2 = 0.105	R1 = 0.045<br>wR2 = 0.178	R1 = 0.041<br>wR2 = 0.156
Largest diff. peak and hole [e·Å^{-3}]	0.28 and -0.22	0.30 and -0.26	0.33 and -0.23	0.28 and -0.21	0.25 and -0.25	0.35 and -0.25
**CCDC number**	**2143620**	**2143614**	**2143617**	**2143615**	**2143619**	**216092**

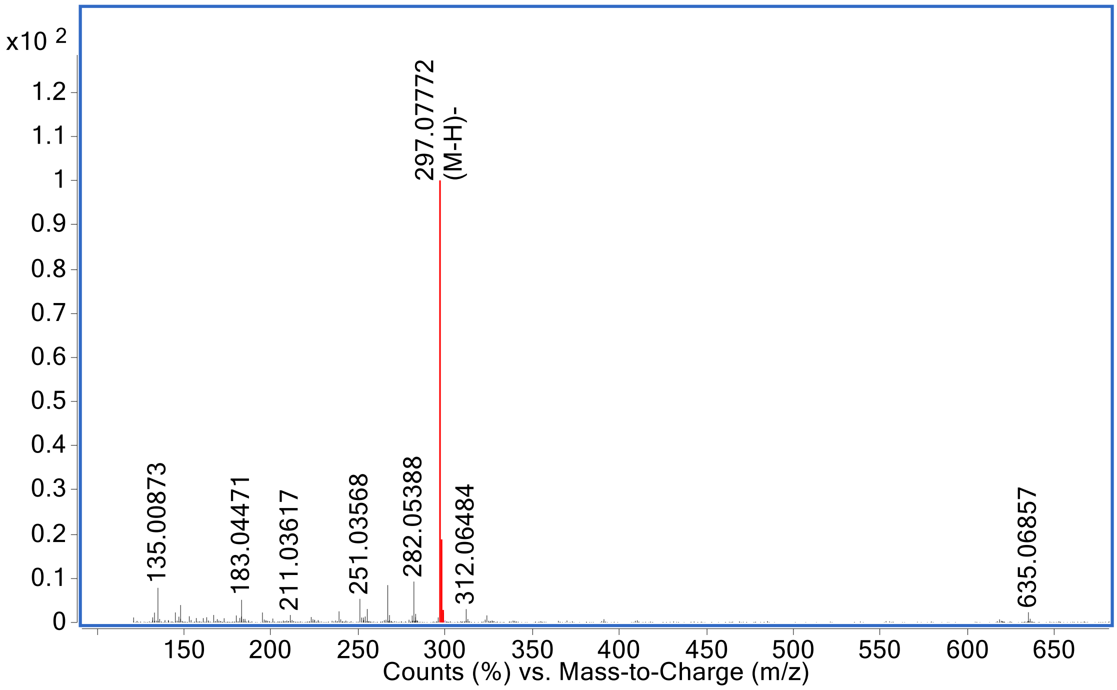

Figure S1. Mass spectrum of compound 4 in negative ion mode (fragmentor = 320 V).

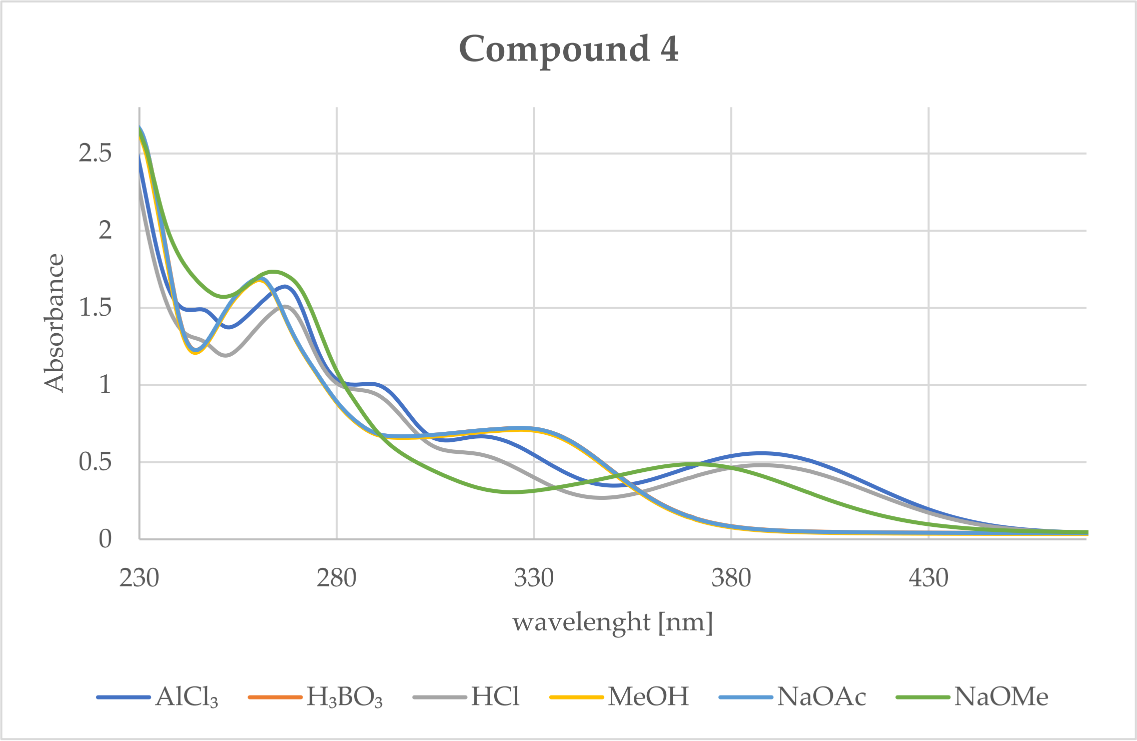

Figure S2. UV spectrum of compound 4.

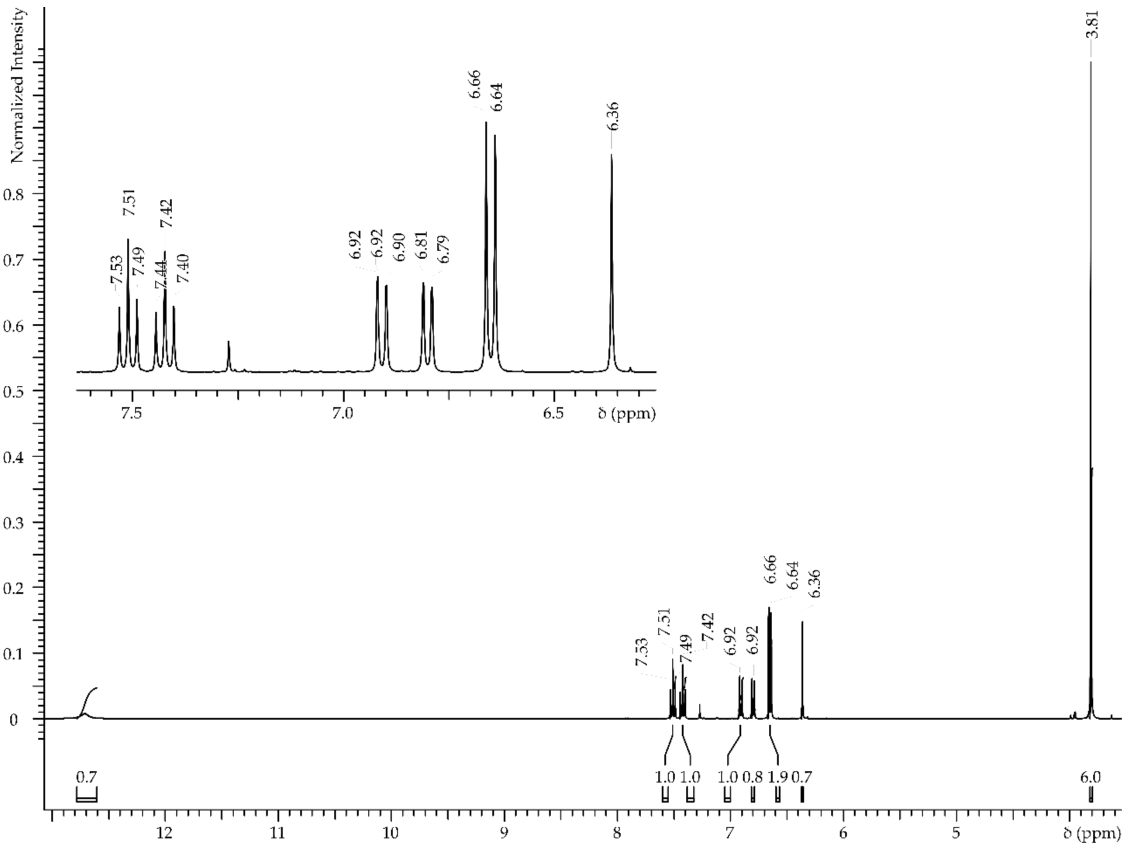

Figure S3. ^{1}H NMR spectrum (400 MHz) of compound 4 in $CDCl_3$.

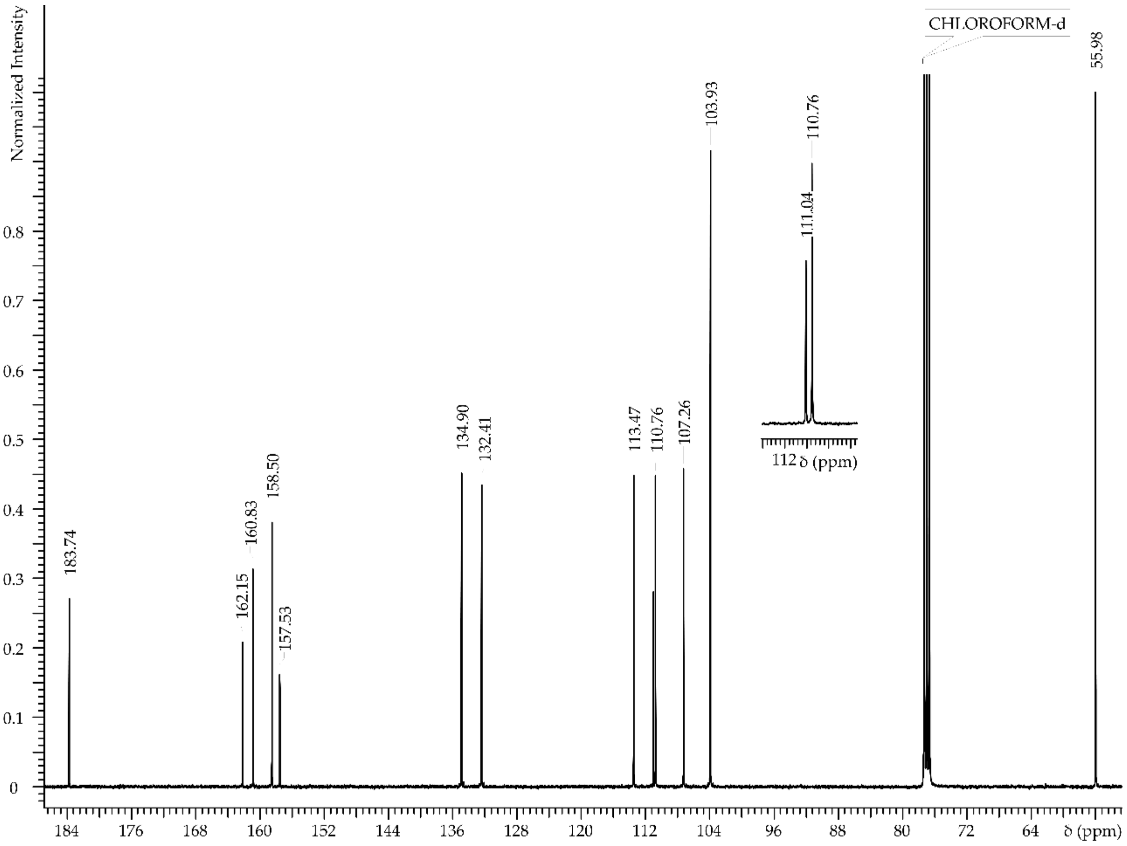

Figure S4. ^{13}C NMR spectrum (400 MHz) of compound 4 in $CDCl_3$.

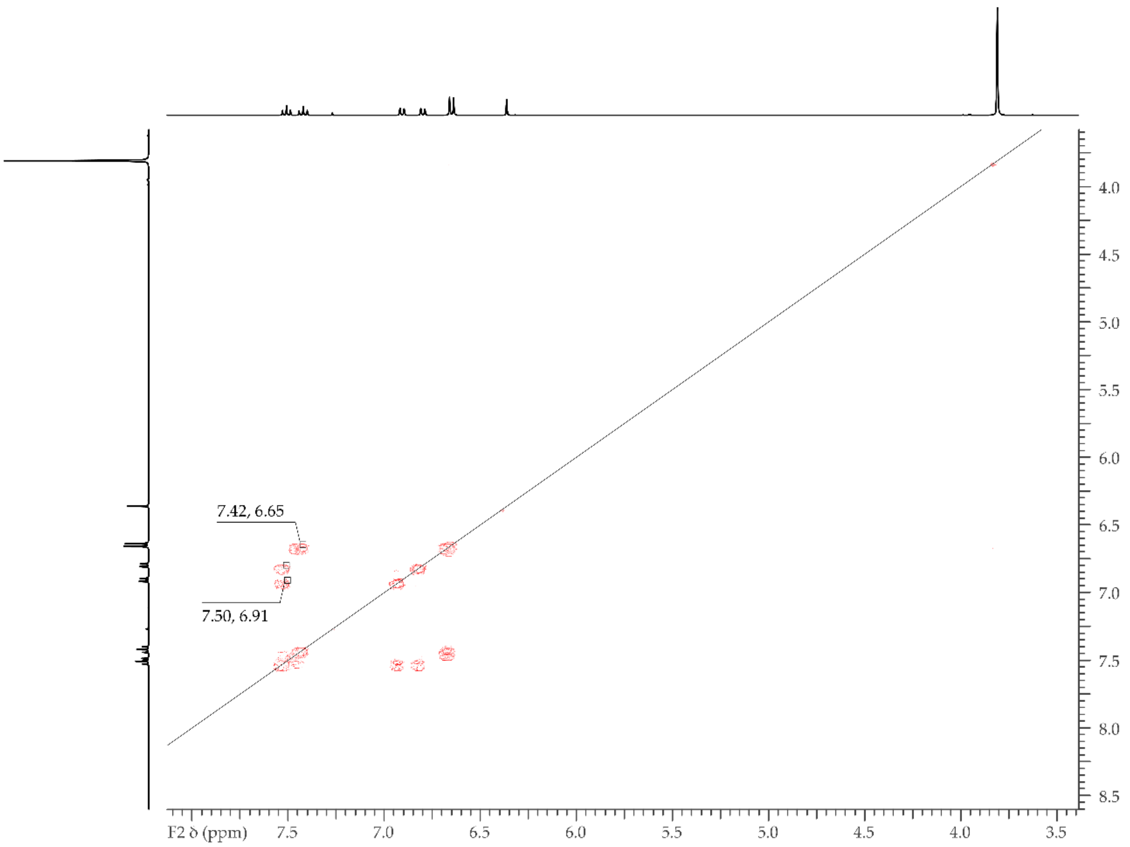

Figure S5. COSY spectrum of compound 4 in $CDCl_3$.

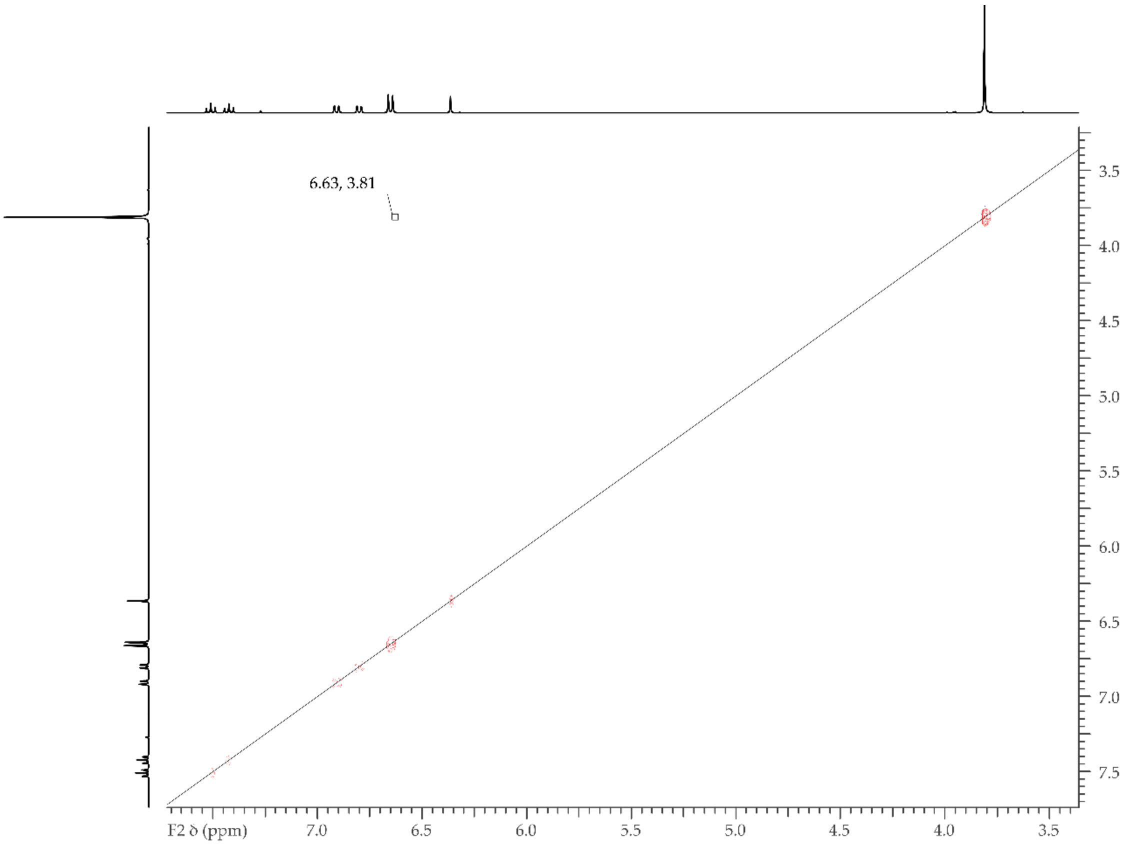

Figure S6. ROESY spectrum of compound 4 in $CDCl_3$.

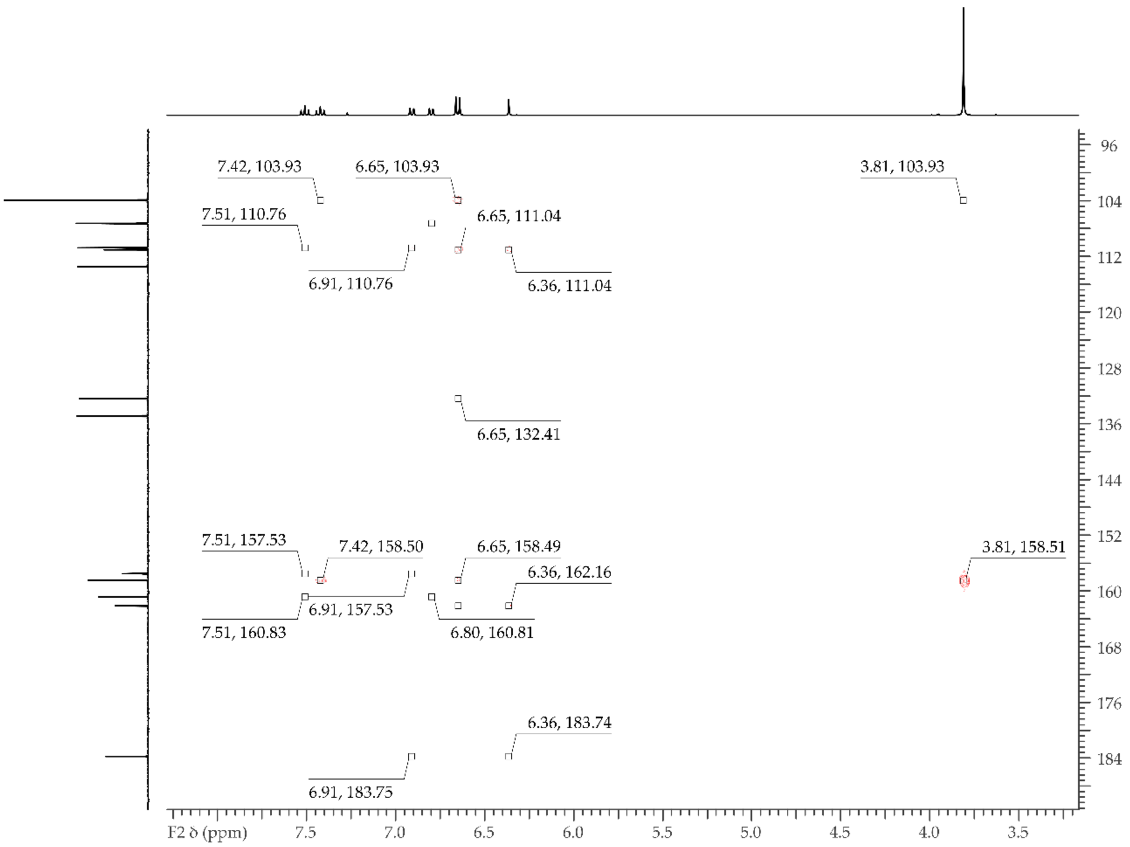

Figure S7. HMBC spectrum of compound 4 in $CDCl_3$.

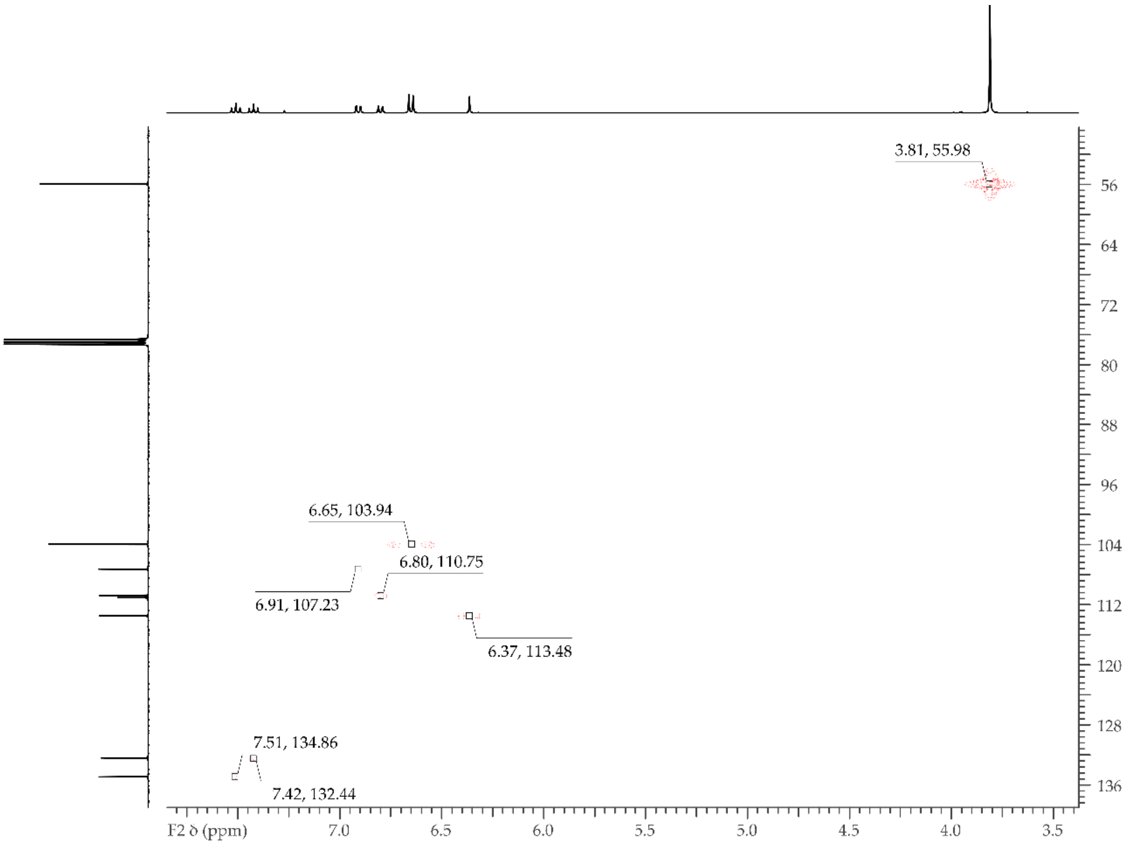

Figure S8. HMQC spectrum of compound 4 in $CDCl_3$.

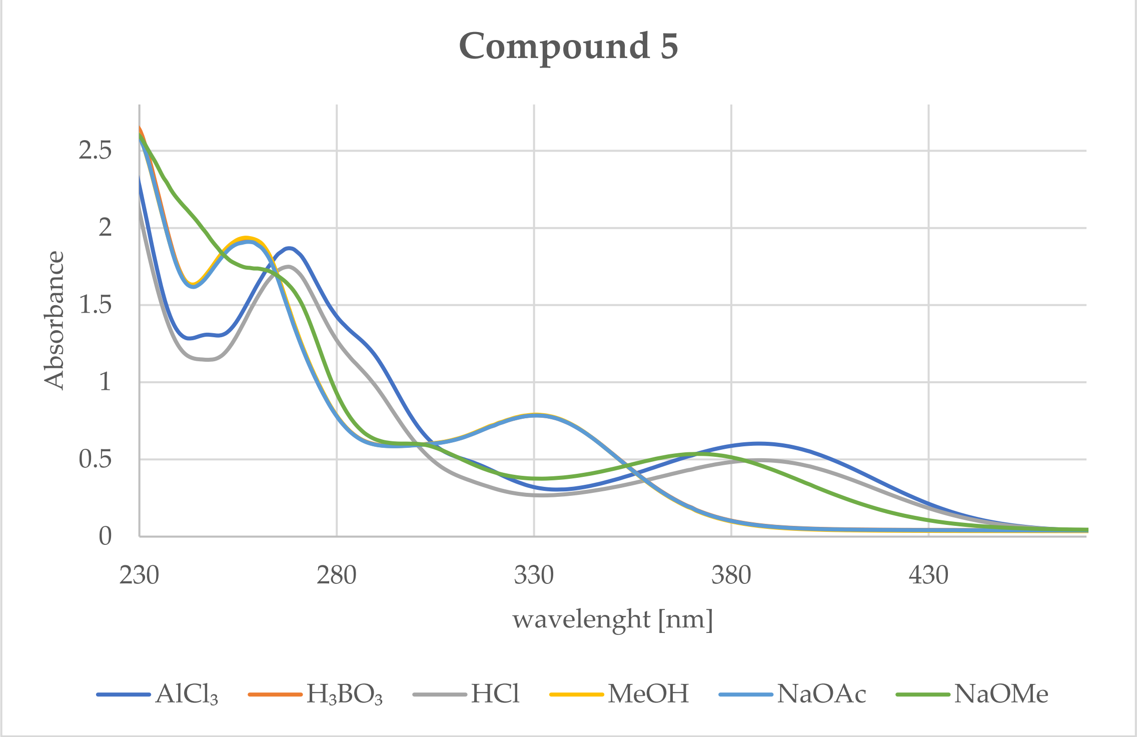

Figure S9. UV spectrum of compound 5.

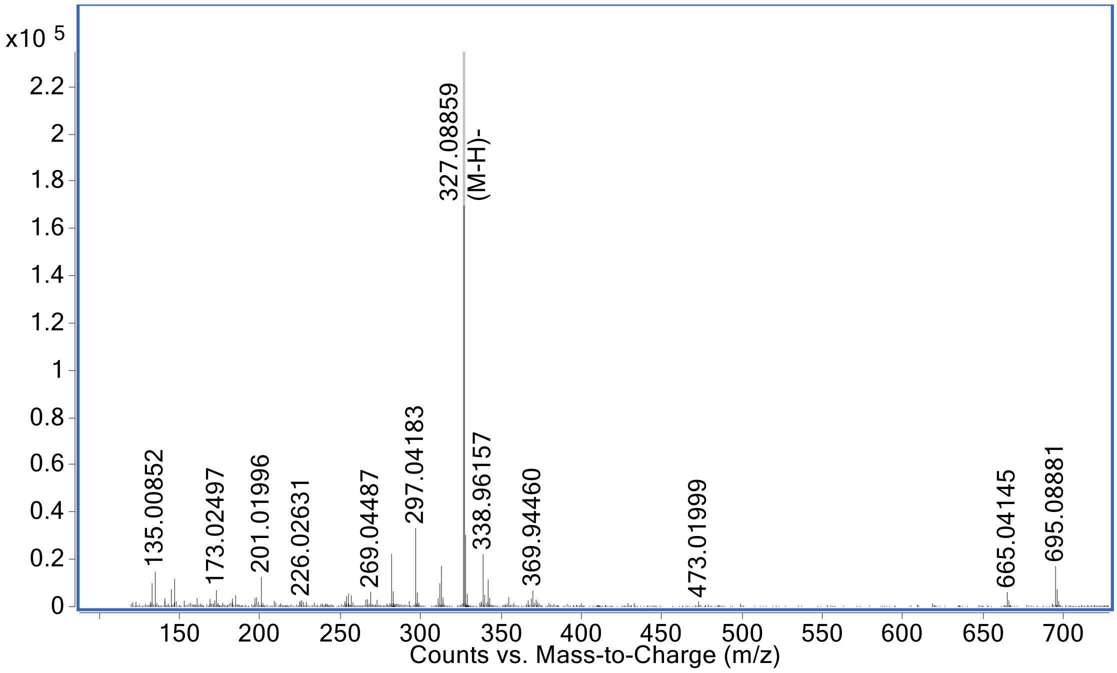

Figure S10. Mass spectrum of compound 5 in negative ion mode (fragmentor = 320 V).

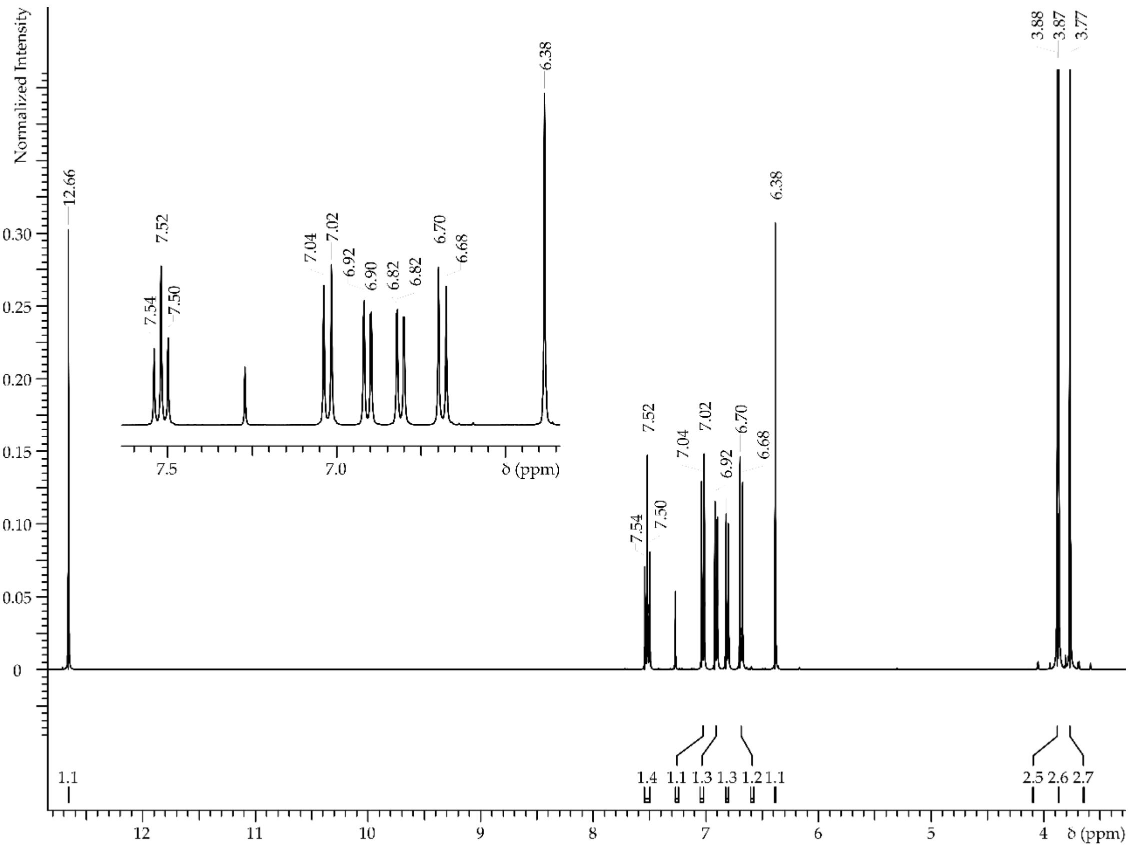

Figure S11. 1H NMR spectrum (400 MHz) of compound 5 in CDCl3.

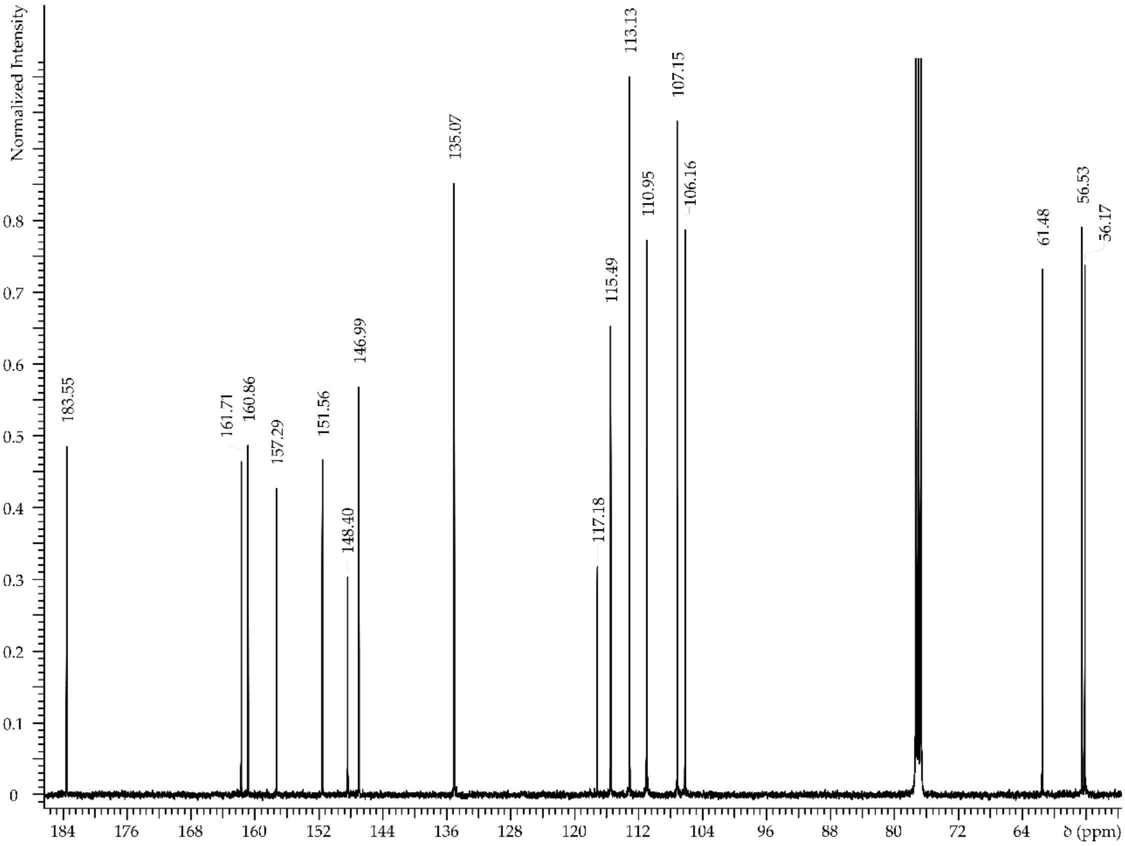

Figure S12. 13C NMR spectrum (100 MHz) of compound 5 in CDCl3.

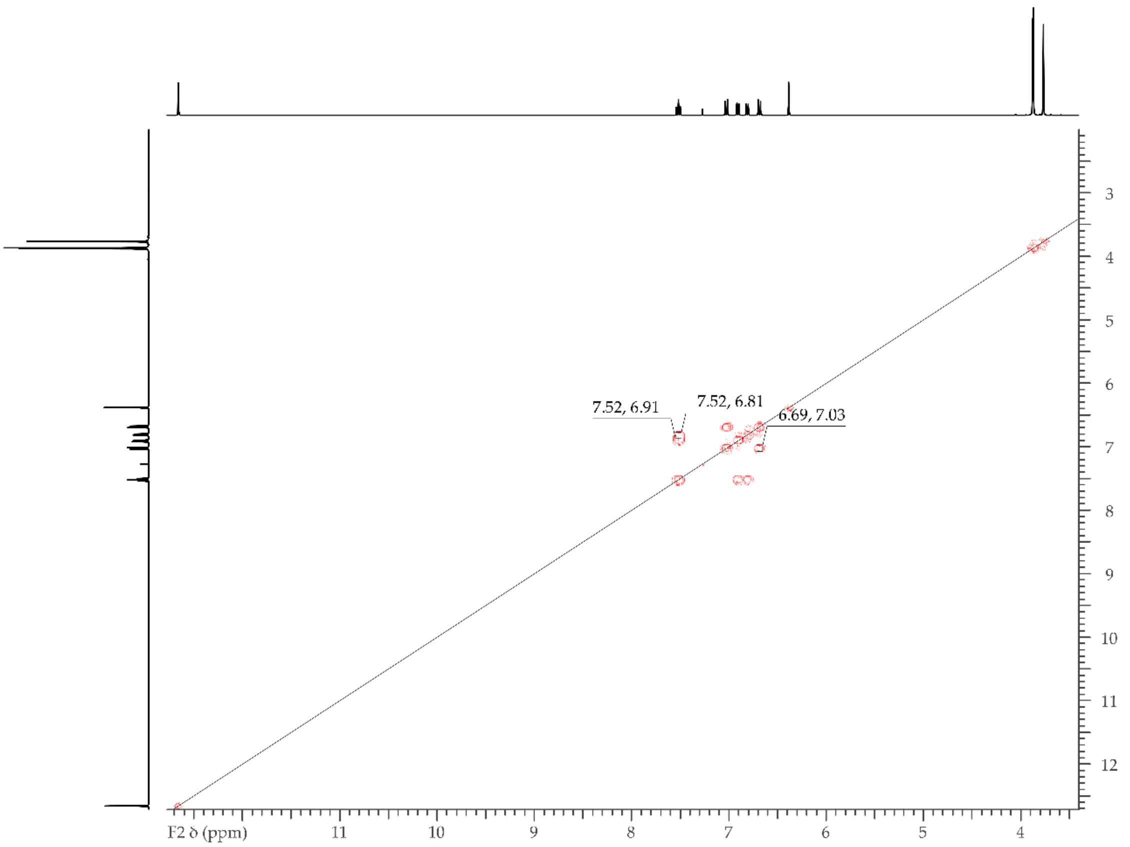

Figure S13. COSY spectrum of compound 5 in $CDCl_3$.

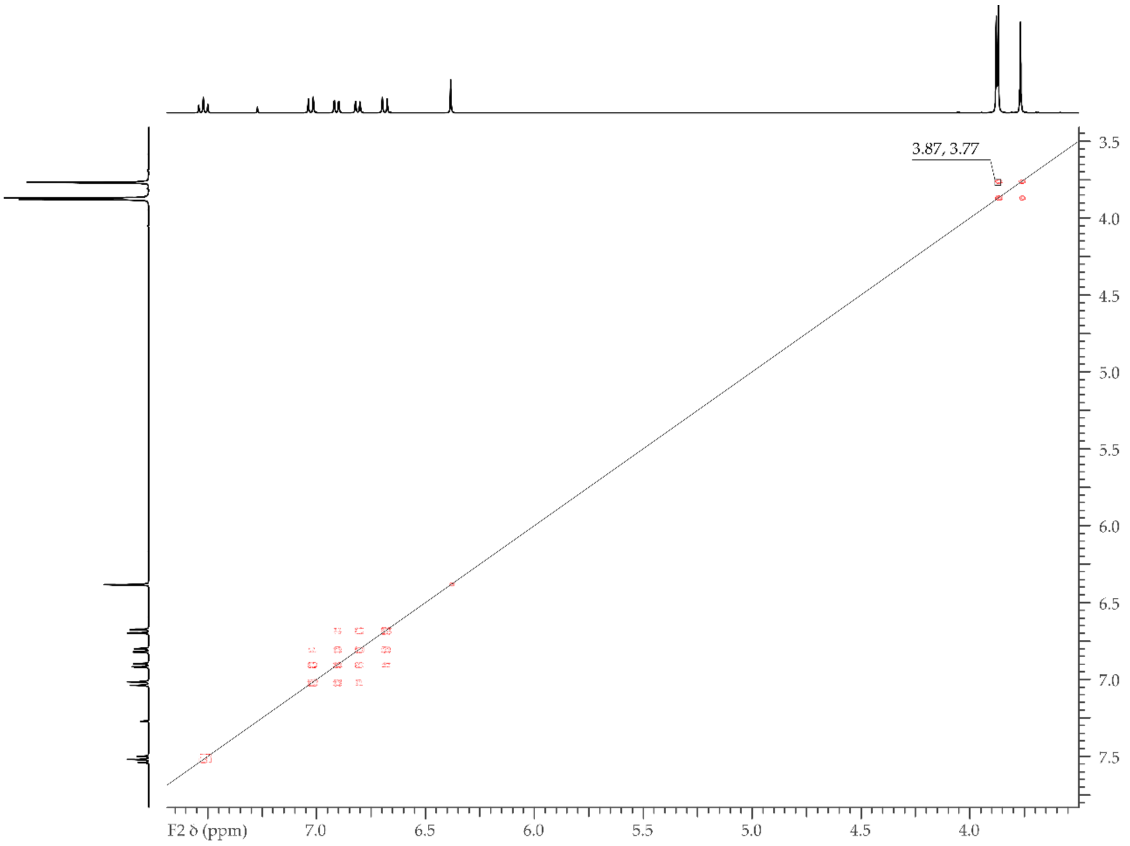

Figure S14. ROESY spectrum of compound 5 in $CDCl_3$.

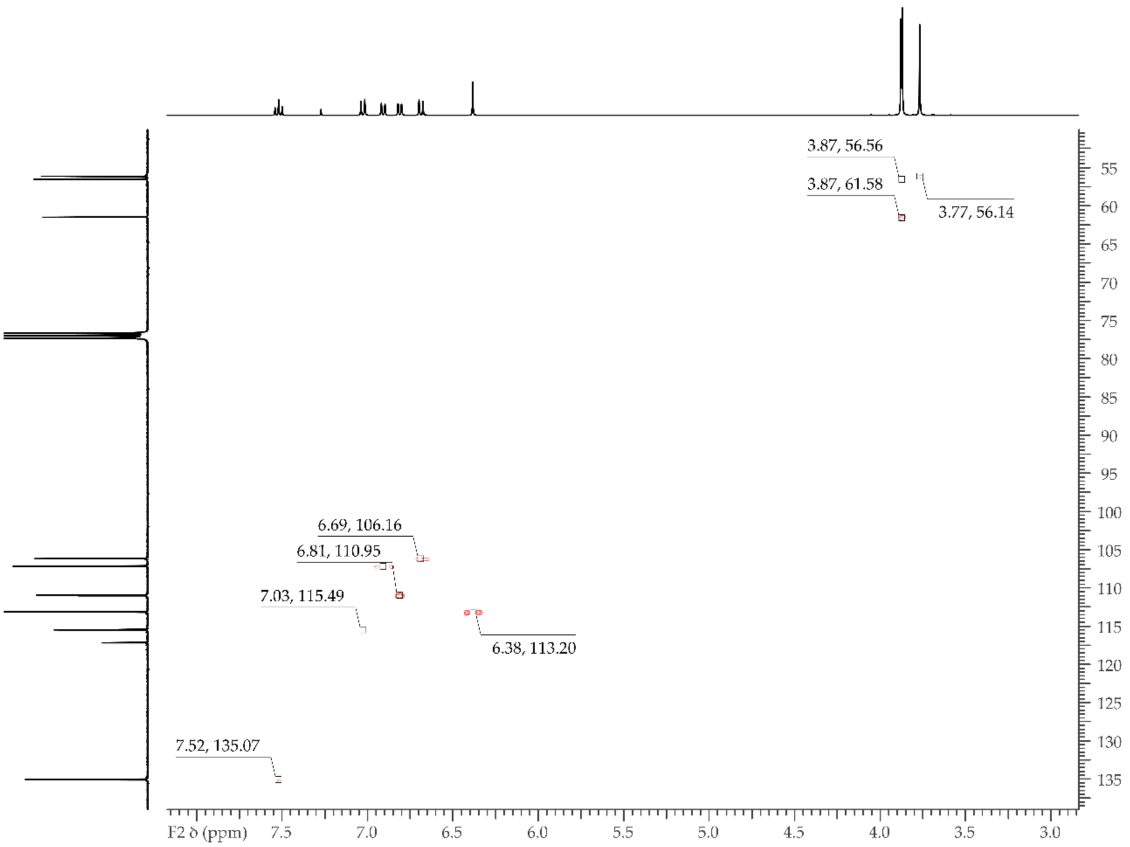

Figure S15. HSQC spectrum of compound 5 in $CDCl_3$.

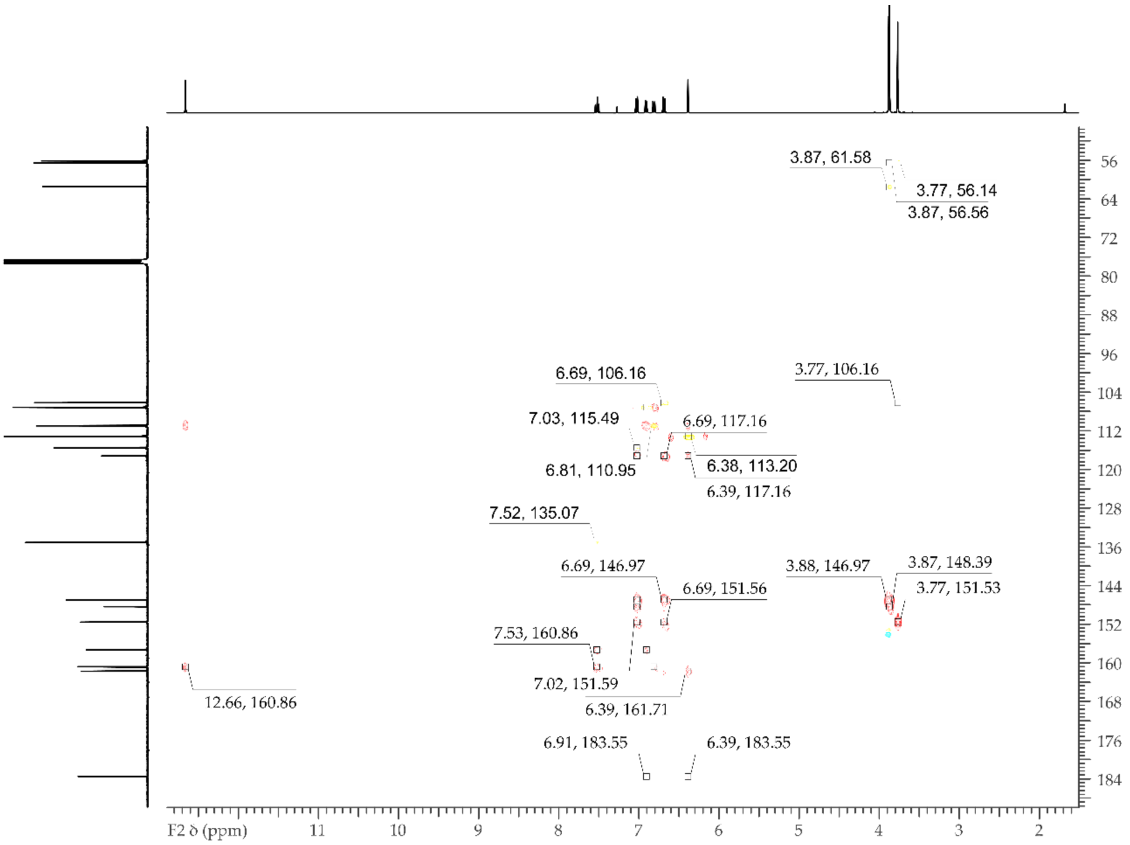

Figure S16. HMBC spectrum of compound 5 in $CDCl_3$.

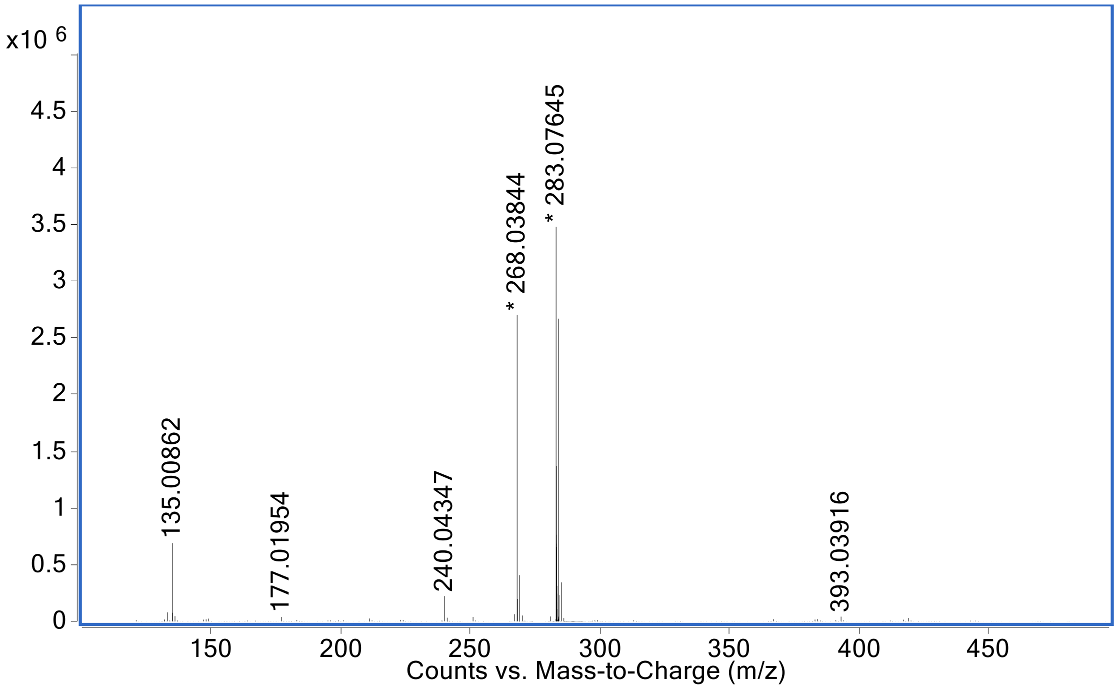

Figure S17. Mass spectrum of compound 6 in negative ion mode (fragmentor = 180 V).

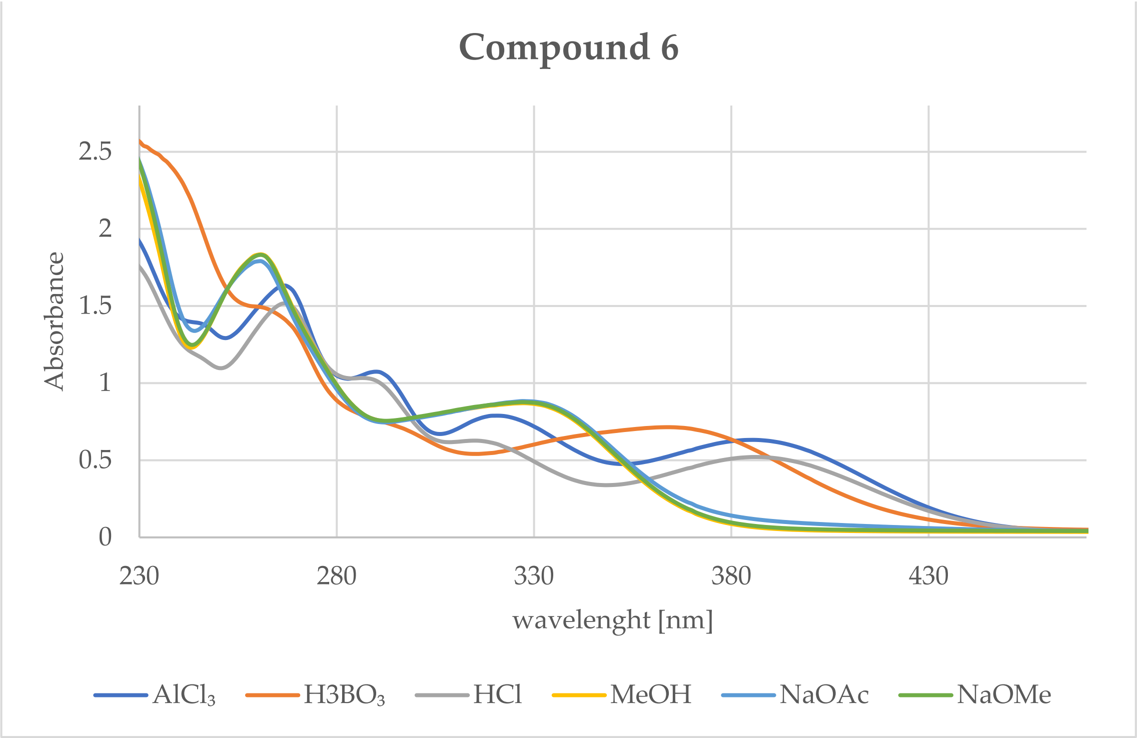

Figure S18. UV spectrum of compound 6.

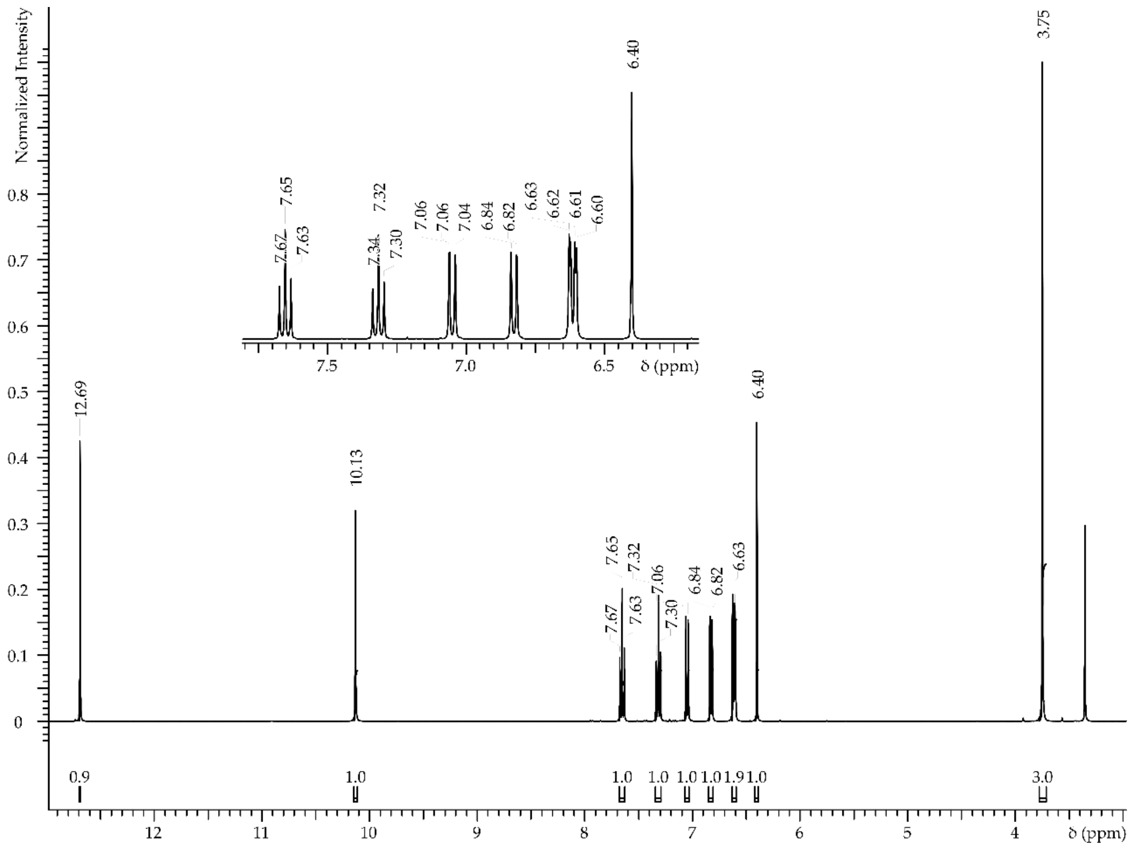

Figure S19. ^{1}H NMR spectrum (400 MHz) of compound 6 in DMSO-*d6*.

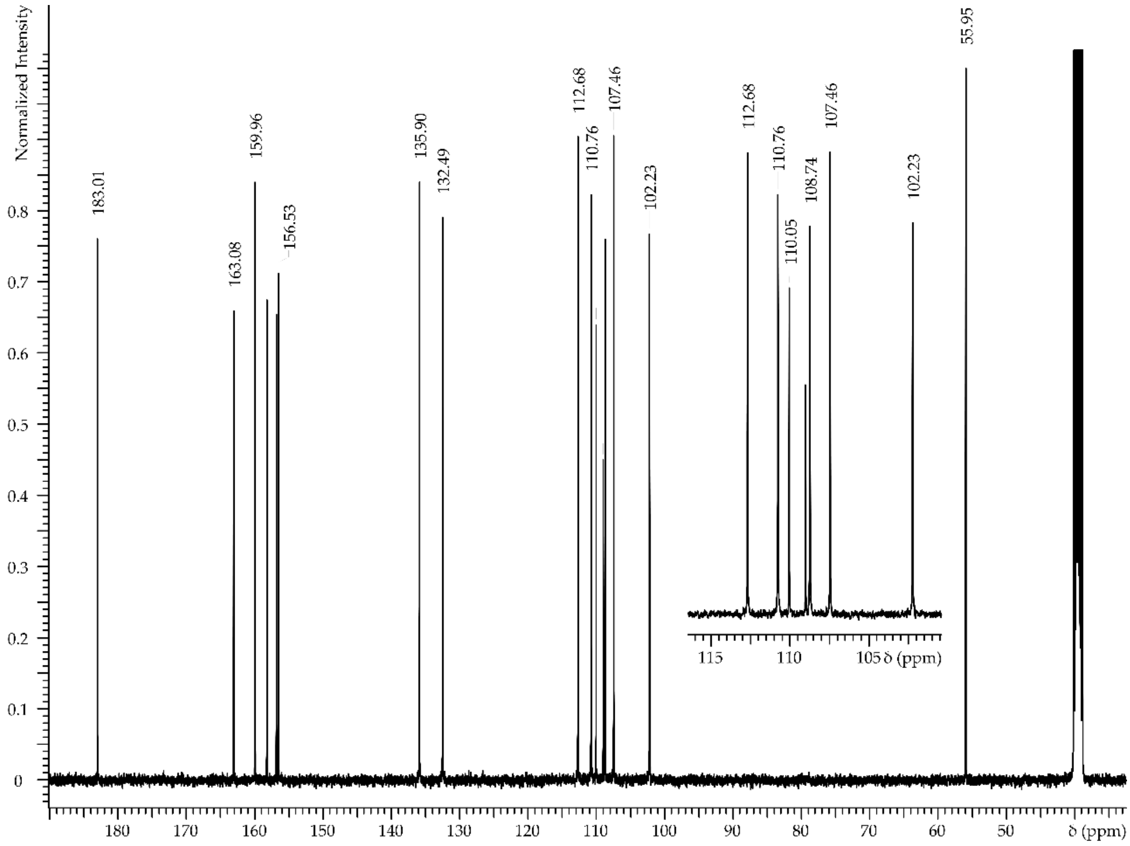

Figure S20. ^{13}C NMR spectrum (100 MHz) of compound 6 in DMSO-*d6*.

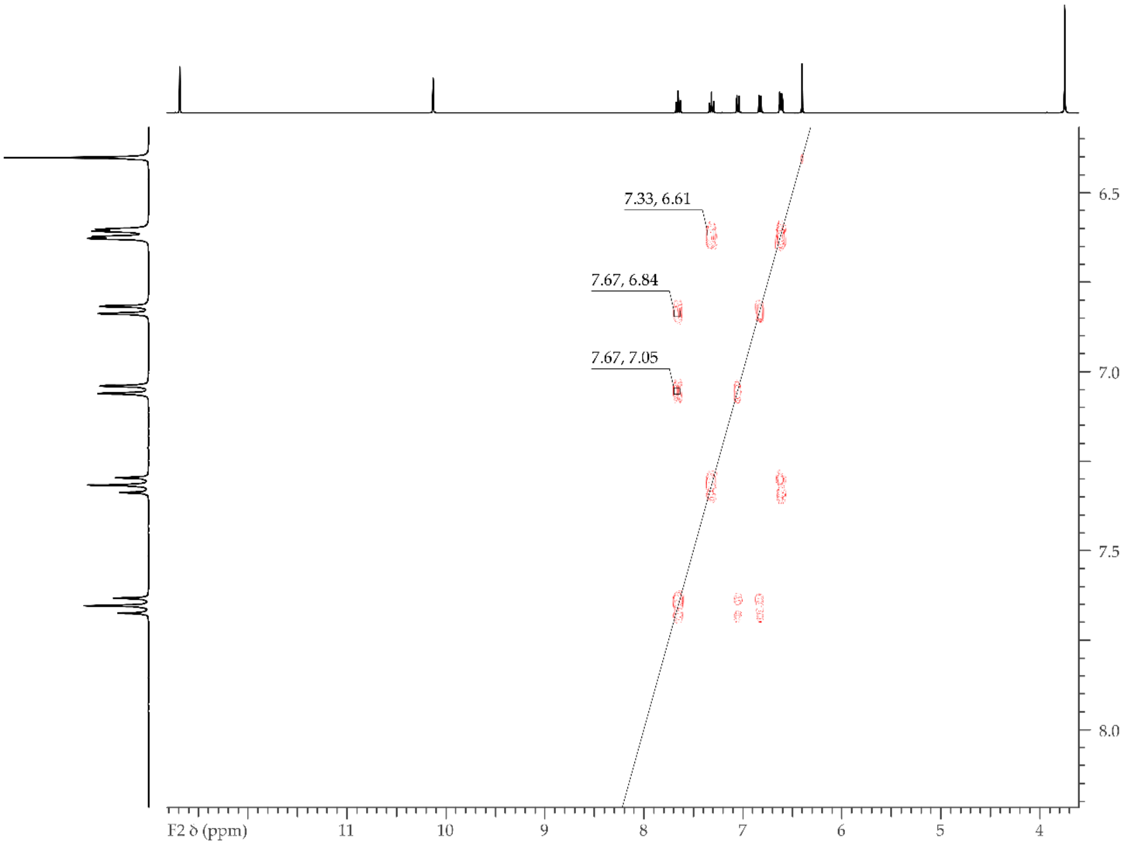

Figure S21. COSY spectrum of compound 6 in DMSO-*d6*.

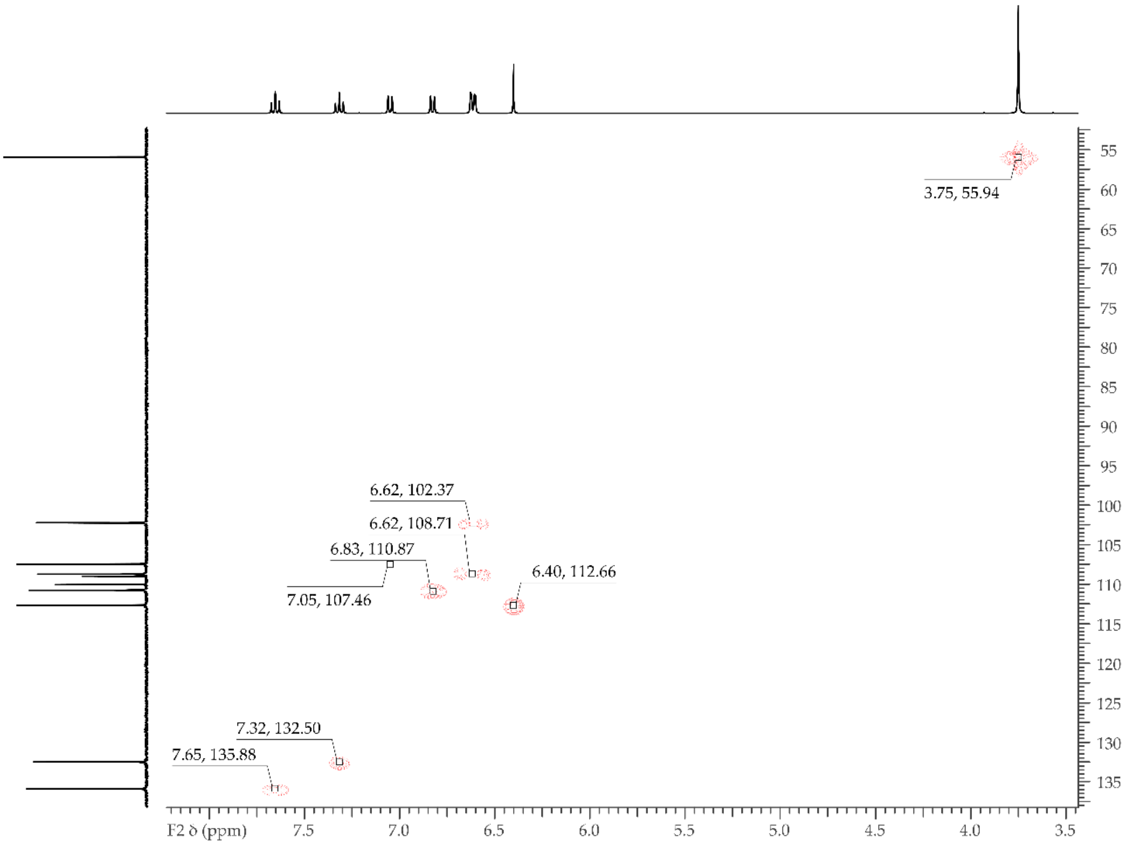

1

Figure S22. HMQC spectrum of compound 6 in DMSO-*d6*.

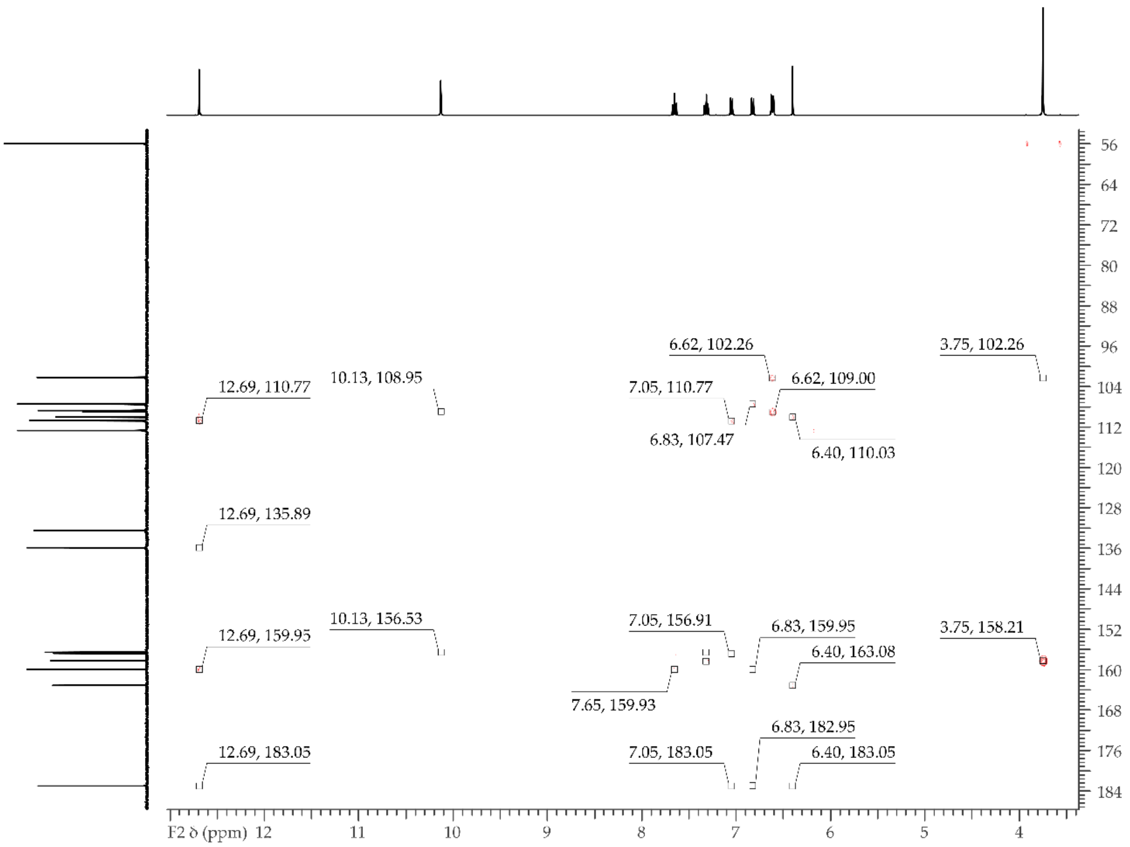

Figure S23. HMBC spectrum of compound 6 in DMSO-*d6*.

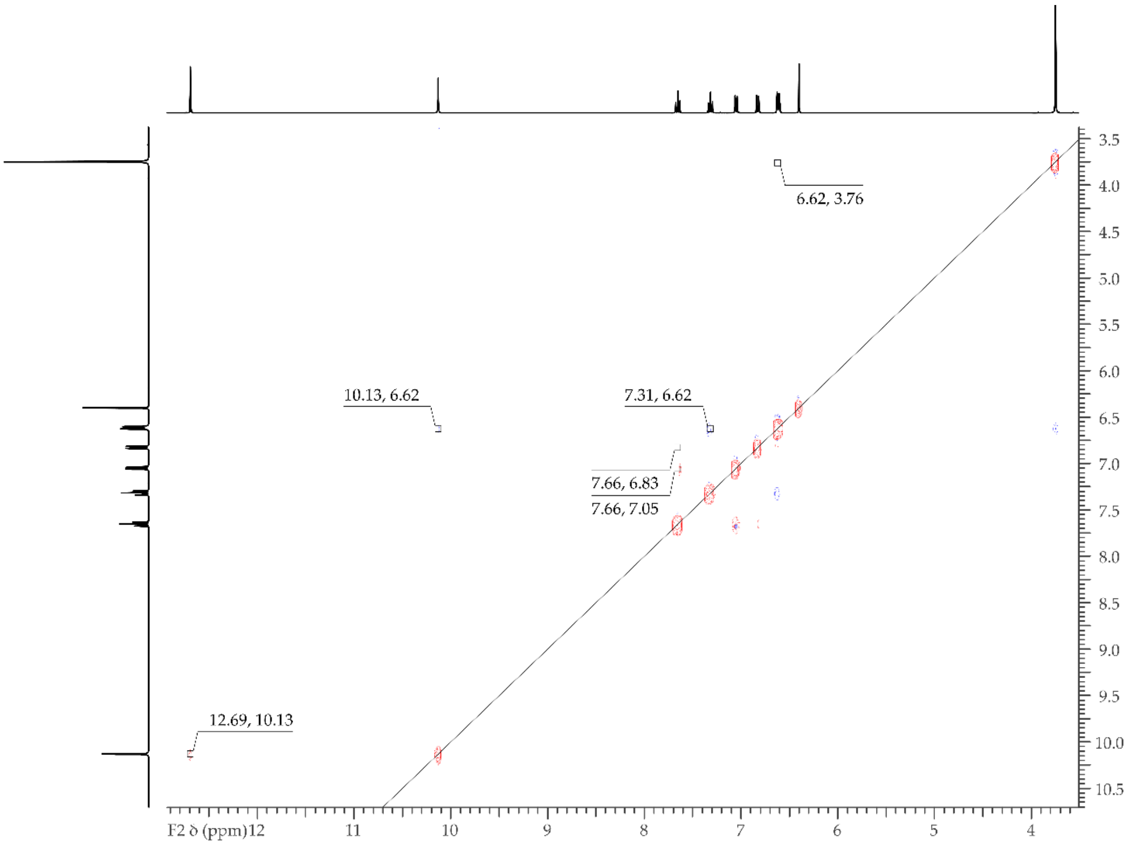

Figure S24. ROESY spectrum of compound 6 in DMSO-*d6*.

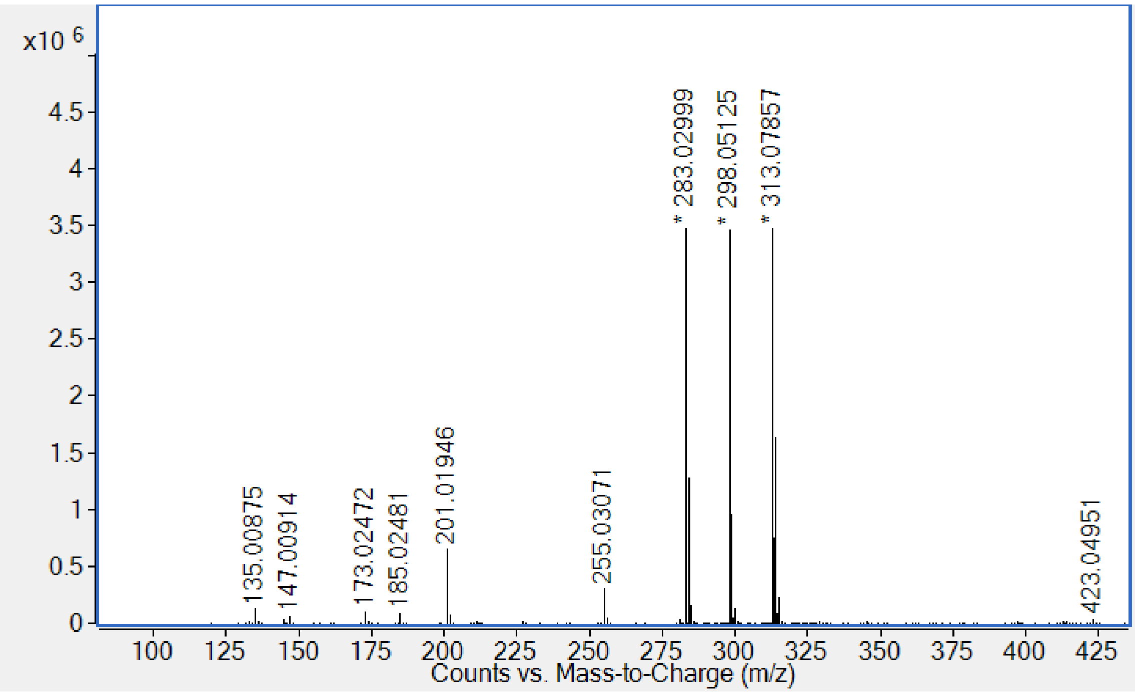

Figure S25. Mass spectrum of compound 7 in negative ion mode (fragmentor = 180 V).

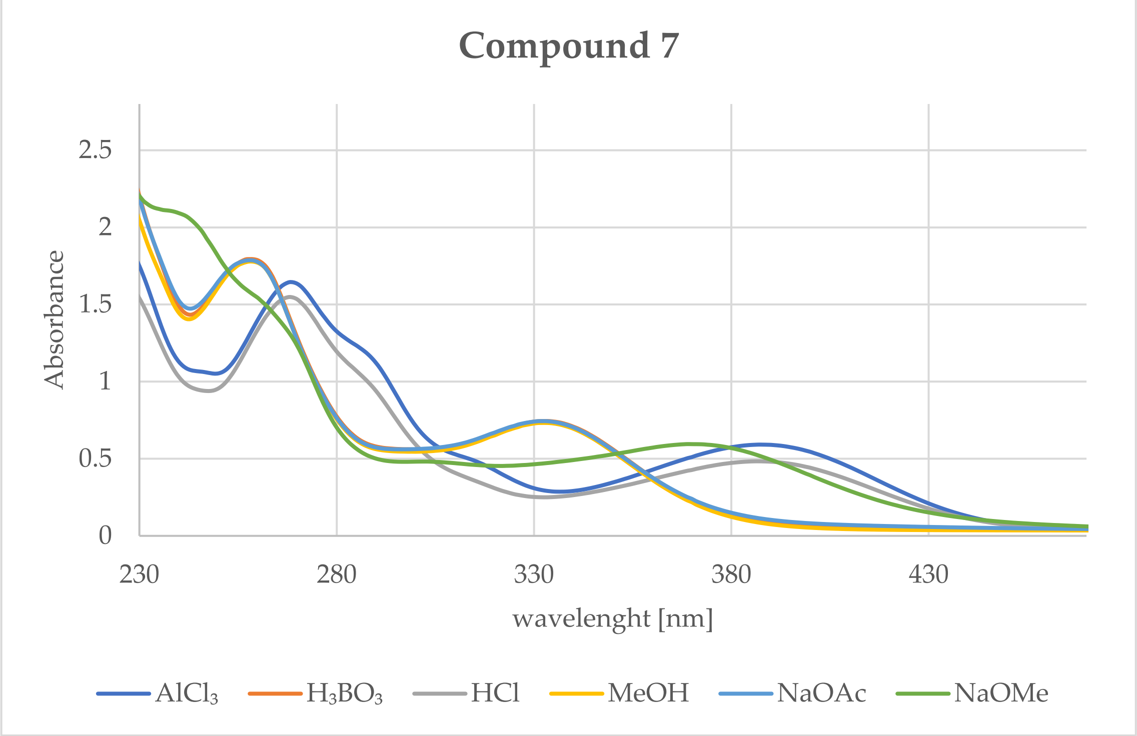

Figure S26. UV spectrum of compound 7.

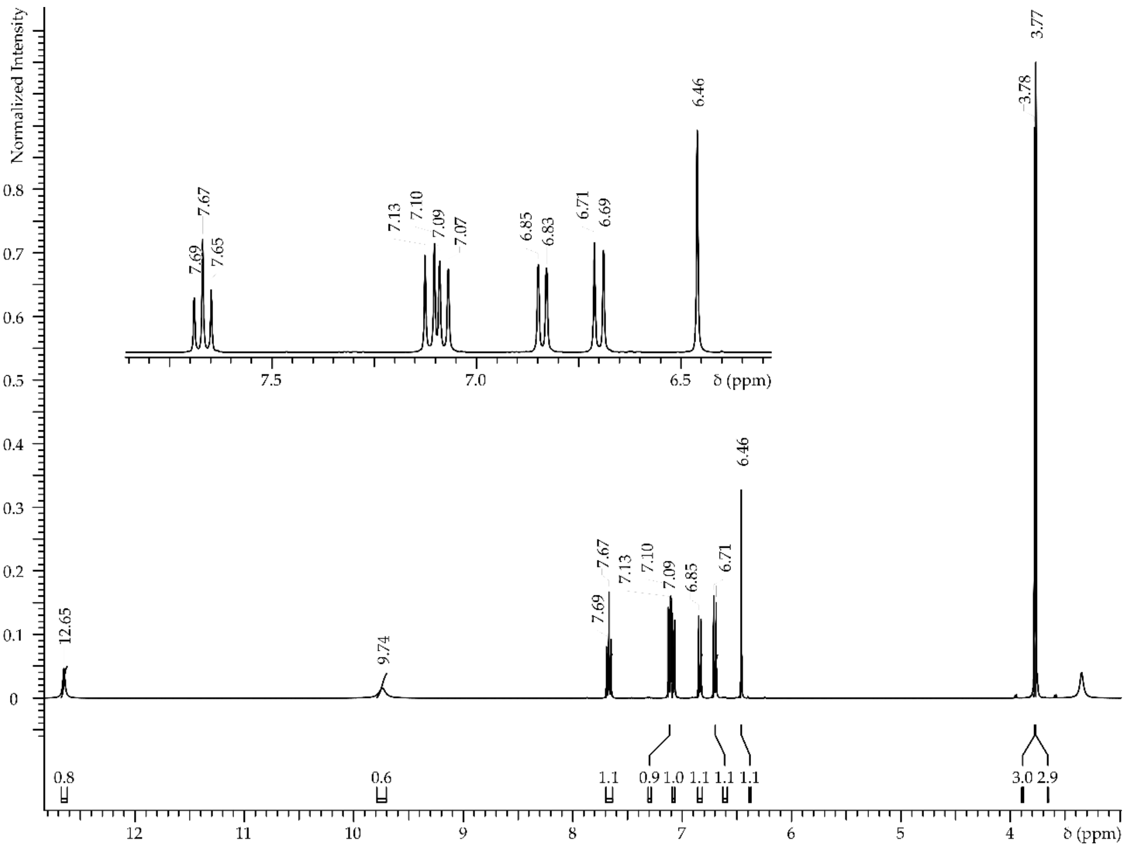

Figure S27. ^{1}H NMR spectrum (400 MHz) of compound 7 in DMSO-*d6*.

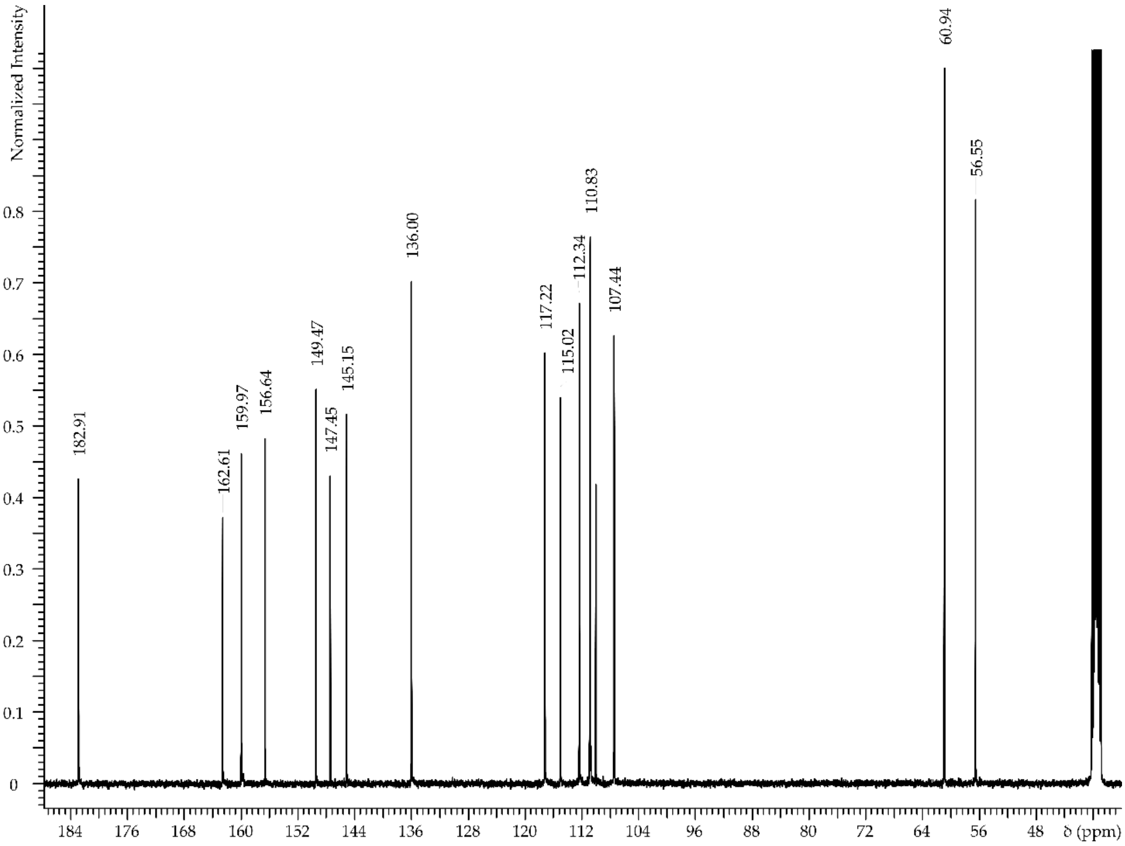

Figure S28. ^{13}C NMR spectrum (100 MHz) of compound 7 in DMSO-*d6*.

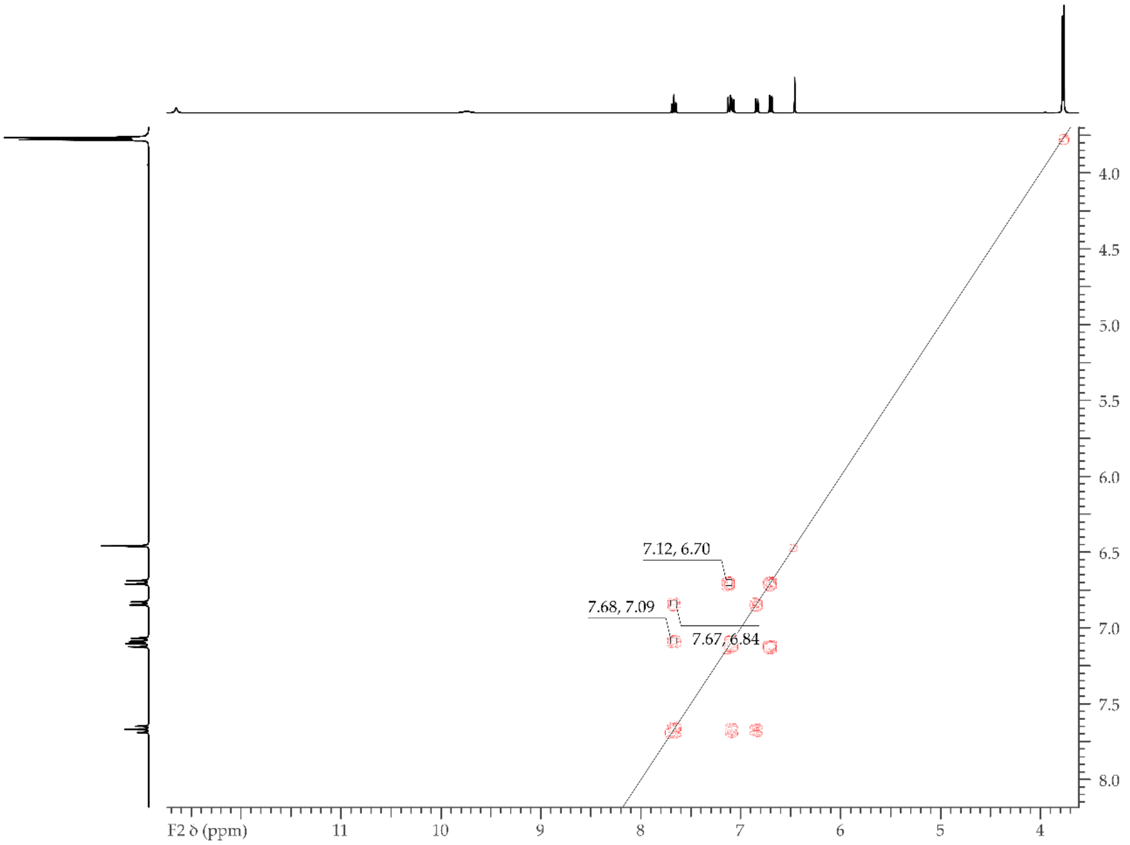

Figure S29. COSY spectrum of compound 7 in DMSO-*d6*.

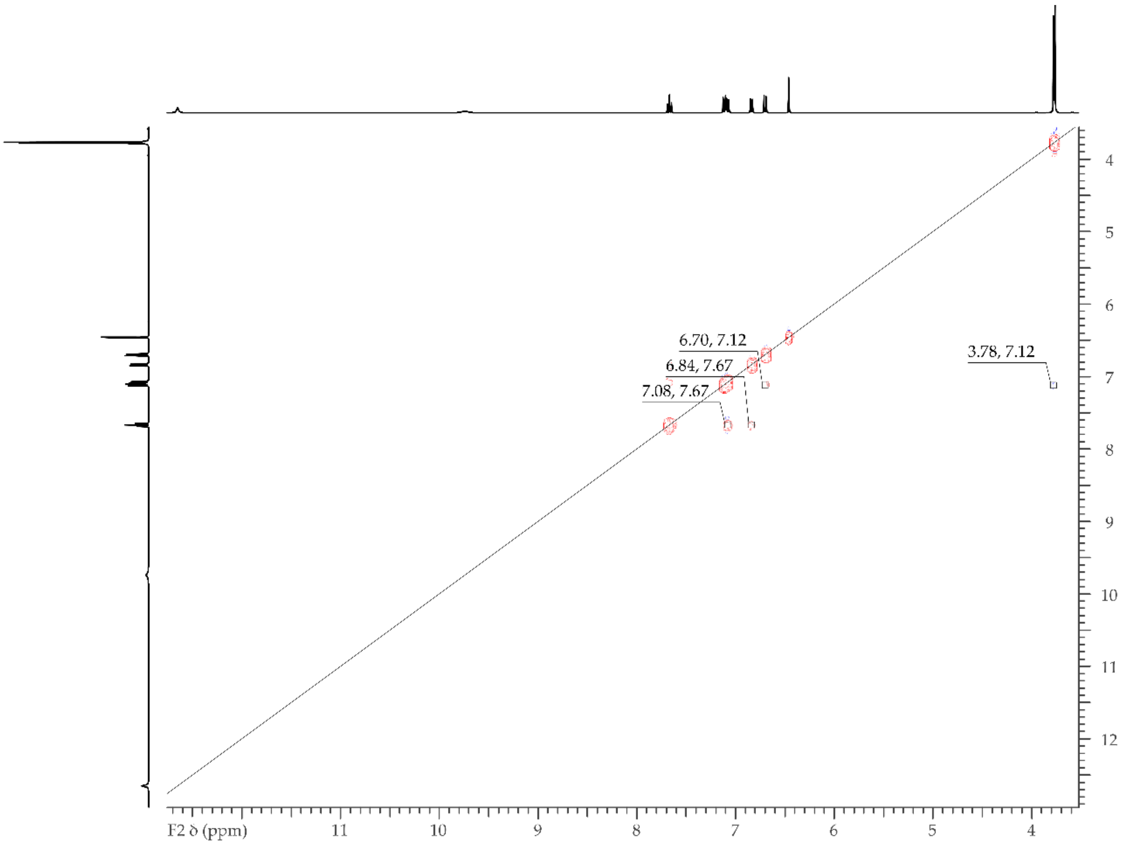

Figure S30. ROESY spectrum of compound 7 in DMSO-d6.

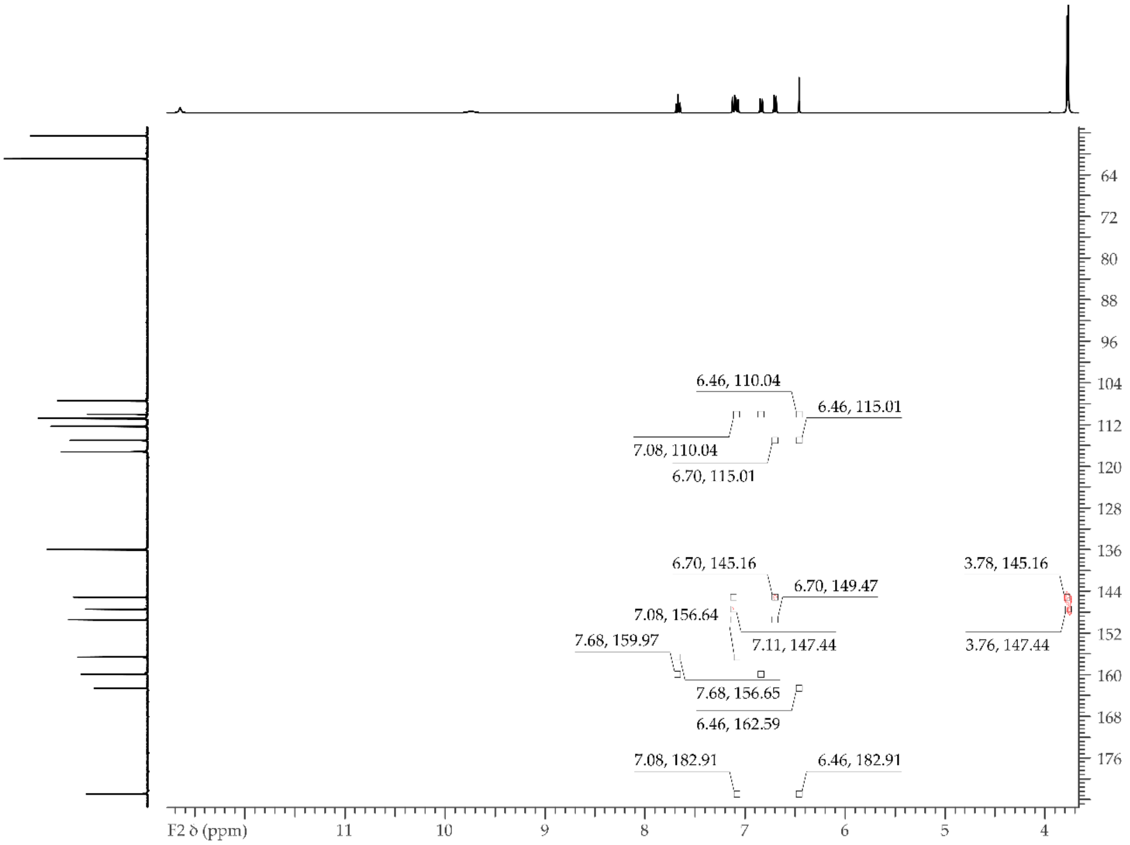

Figure 31. S: HMBC spectrum of compound 7 in DMSO-*d6*.

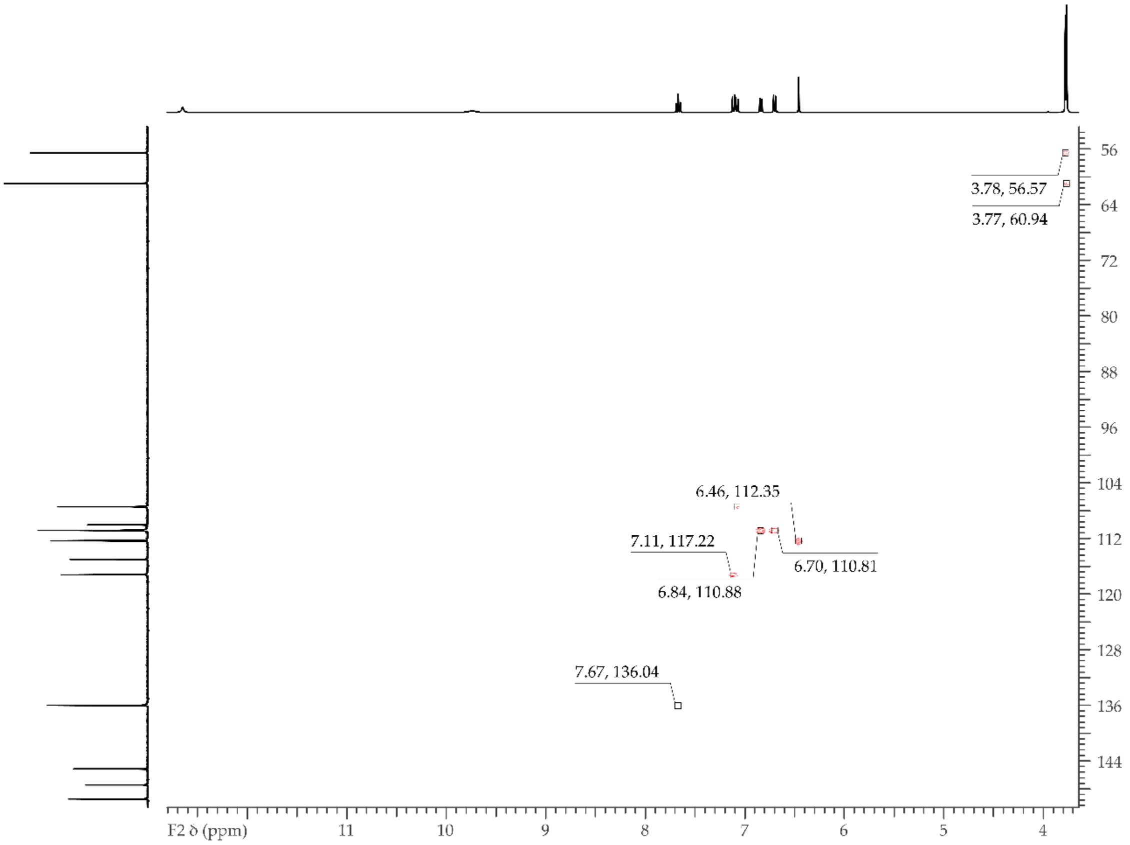

Figure 32. S: HSQC spectrum of compound 7 in DMSO-*d6*.

Table S2. Main HPLC optimization method and validation parameters.

Parameter	Compounds							
	**1**	**2**	**3**	**4**	**5**	**6**	**7**	**zap**
Linear range [µg/mL]	1-50	1-50	2-75	1-50	1-50	1-50	1-50	5-150
Regression equation[A,D]	26.551*x* + 0.006	51.555*x* - 1.5942	29.22*x* - 3.3111	23.949*x* + 0.0299	20.825*x* - 0.1623	23.529*x* + 1.35	21.619*x* + 4.0224	18.4*x* - 1.4962
$R^{2\#}$	0.9999	0.9999	0.9999	0.9999	0.9999	0.9999	0.9999	0.9999
LOD [µg/mL]	0.4	0.4	0.89	0.48	0.44	0.77	0.77	4.94
LOQ [µg/mL]	1.22	1.21	2.71	1.46	1.34	2.33	2.33	14.99
Accuracy [%][B]	100.75 ± 3.27	99.26 ± 2.31	100.46 ± 1.63	100.85 ± 2.81	100.98 ± 3.31	100.82 ± 3.89	99.76 ± 1.83	99.86 ± 0.86
Intraday precision [%][B]	0.82	0.81	0.67	0.88	0.74	0.82	0.75	0.71
Interday precision [%][B]	1.01	0.95	1.19	1.22	0.9	1.29	1.14	1.09

[A] the value for y corresponds to the peak area and x to the concentration, respectively; [B] expressed as mean with coefficient of variation value.

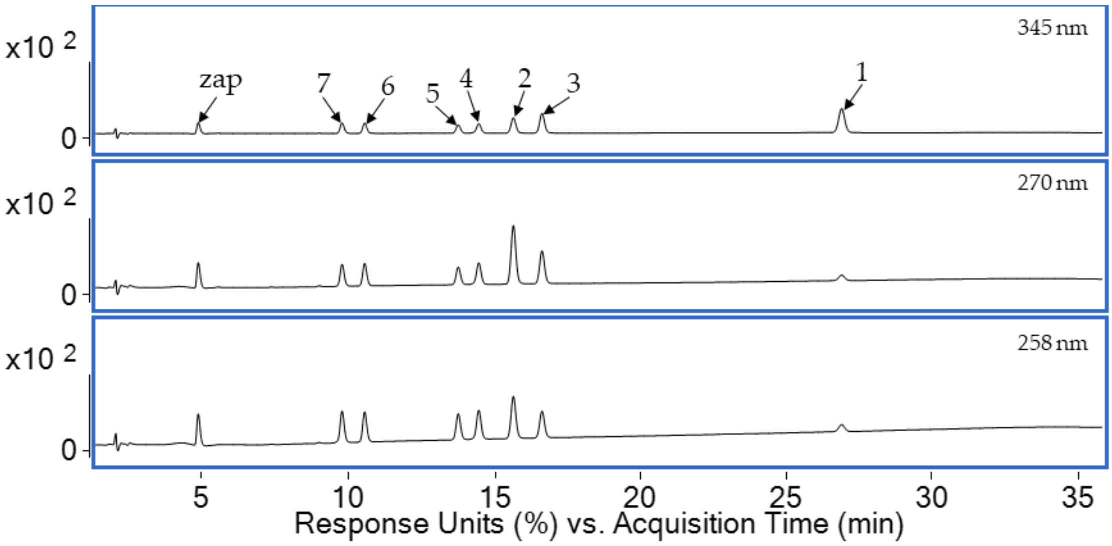

Figure S33. UV–VIS chromatogram of separated compounds 1-7 and ZAP obtained by HPLC-PDA.

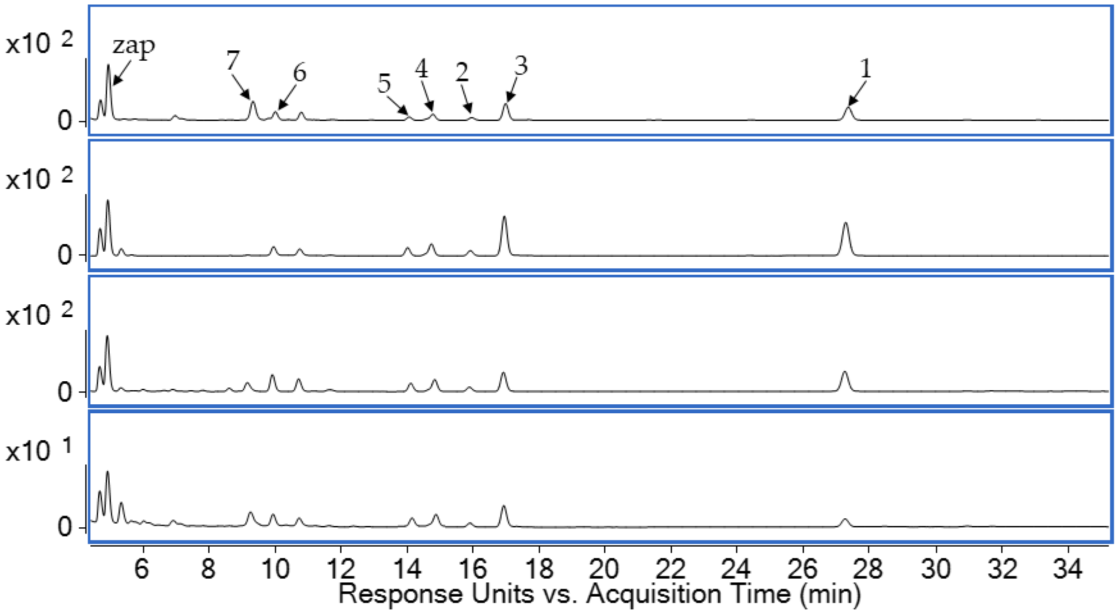

Figure S34. UV–VIS chromatogram of extracts HP1 and HP6-8 with well-separated compounds 1-7 and zapotin (ZAP) obtained by HPLC-PDA (345 nm). .
